# Zwitterionic Modification of PSMA Ligands Reduces Off-Target Binding and Tissue Retention

**DOI:** 10.1021/acs.jmedchem.5c03849

**Published:** 2026-03-13

**Authors:** Lennart F. V. Spickschen, Roland Thünauer, Aleksander J. Swierzewski, John M. Van Wazer, Amanda Fears, Matthew D. Silva, Daniel L. J. Thorek, Elke Oetjen, Wolfgang Maison

**Affiliations:** a Department of Chemistry, Institute of Pharmacy, University of Hamburg, Bundesstrasse 45, Hamburg 20146, Germany; b Technology Platform Light Microscopy (TPLM), University of Hamburg and Advanced Light and Fluorescence Microscopy (ALFM) Facility, Centre of Structural Systems Biology, Notkestrasse 85, Hamburg 22607, Germany; c Department of Radiology, Washington University, School of Medicine, 510 S. Kingshighway Blvd., Saint Louis, Missouri 63110, United States; d EMIT Imaging, Inc. 12 Michigan Drive, Natick, Massachusetts 01760, United States; e Institute of Clinical Pharmacology and Toxicology, University Medical Center Hamburg-Eppendorf, Martinistrasse 52, Hamburg 20246, Germany

**Keywords:** PSMA targeting, prostate cancer, PSMA-617, zwitterions, off-target toxicity

## Abstract

Off-target tissue retention is a serious limitation for prostate-specific membrane antigen (PSMA)-targeted drugs. This study addresses key questions regarding the role of zwitterionic modifications in PSMA-ligand design for tissue distribution. A series of fluorescent PSMA-ligands was synthesized and evaluated with respect to PSMA-binding, tumor uptake, and biodistribution in cell experiments and in mice. The data revealed that the introduction of two zwitterionic groups into the linker domain of the PSMA-specific conjugates was particularly advantageous. The resulting compound **10** combined high and specific PSMA-binding affinity (IC_50_ = 4.39 ± 1.69 nM) and good uptake in tumor cells and tumor xenografts with extremely low off-target tissue retention. A major practical advantage of this strategy is its simple synthetic realization using solid-phase peptide synthesis with commercial building blocks and their modification using click-chemistry. Zwitterionization is therefore easily transferable to other targeting vectors and alternative effector molecules, for example in radiopharmaceuticals.

## Graphical abstract

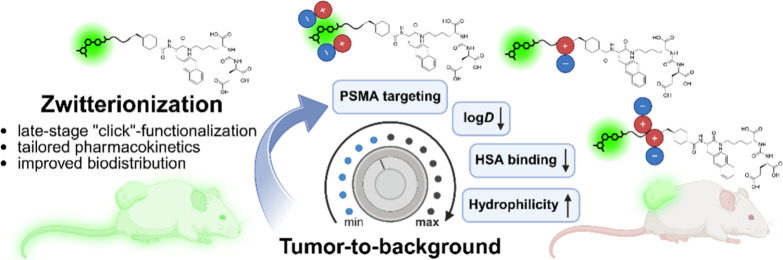

## Introduction

Prostate cancer is the second most common cancer and fifth leading cause of cancer-related death among men with incidence rates expected to rise worldwide.^1^ For patients with aggressive and treatment-resistant disease, such as metastatic castration-resistant prostate cancer, prognosis has long been poor.^2^ In recent years, prostate specific membrane antigen (PSMA)-targeted imaging and therapy have emerged as valuable clinical tools complementing standard therapies such as chemotherapy and external beam radiotherapy.^3,4^ The radioactive compounds [^177^Lu]­Lu-PSMA-617 for endoradiotherapy and [^68^Ga]­Ga-PSMA-11 for PET imaging have been approved for clinical use.^5,6^ PSMA-targeted near-infrared (NIR)-fluorescent derivatives have not been approved yet, but they are highly promising for application in fields like intraoperative imaging.^7–15^ However, the currently used PSMA-targeted drugs still face many important limitations with respect to nontarget specific tissue retention, which compromises both diagnostic (signal-to-noise) and therapeutic (off-target toxicity) applications.

Off-target binding can be caused by the physicochemical properties of the PSMA-specific drug or the expression of PSMA in healthy tissues such as salivary glands and renal proximal tubules.^16–20^ These issues are particularly relevant for targeted radiotherapy, which is severely limited by off-target toxicity causing severe side effects, such as radiation-induced xerostomia,^21^ nephrotoxicity,^22^ and late bone marrow toxicity.^23^ Diagnostic applications of PSMA-specific drugs can also be compromised by off-target retention. The latter reduces the signal-to-background ratio and can thus impair the detection of small lesions.^24^

A variety of strategies has been explored to overcome these challenges,^25^ including the use of long-lived radionuclides to improve lesion contrast,^26^ modifications to extend drug circulation time while enhancing tumor uptake,^27–29^ coadministration of PSMA-ligands for organ-specific PSMA-blockage^30,31^ and addition of d-glutamate residues to reduce salivary gland uptake.^32–34^ The latter approach suggests that ligand optimization can lead to a decreased uptake in nontarget tissues while simultaneously retaining good tumor uptake, even though both off-target tissue and tumor contain PSMA. This tissue differentiation might be a consequence of different pharmacokinetics and/or tissue-specific differences in the target such as glycoforms.^35,36^

A promising but underexplored strategy to reduce off-target retention of drugs involves their “decoration” with zwitterionic moieties. Examples include NIR-dyes with improved biodistribution,^37–42^ development of high-relaxivity contrast agents for MRI,^43–45^ improved chelators for zirconium^46^ and zwitterionic FAP-inhibitors.^47^ It is applied on a broader basis in material science, where surface zwitterionization leads to good biocompatibility and low-fouling properties.^48^ Materials bearing zwitterionic groups such as sulfobetaines and amine *N*-oxides proved to be particularly valuable in this context.^49,50^ Both are highly hydrated kosmotropic groups that increase hydrophilicity and reduce nonspecific interactions of drugs with biomolecules.^51^ Only a few drugs targeted to PSMA incorporating zwitterionic groups have been reported so far. Choi and co-worker reported the first conjugate of a zwitterionic NIR-dye with a KuE (Lys-urea-Glu) targeting vector.^12^ This study was recently complemented with a set of alternative zwitterionic NIR-dyes.^52^ In addition, a PSMA-targeted radioligand, based on a KuE-targeting vector and a phosphobetaine group in the linker moiety has recently been reported.^29^ Finally, Perrin and co-worker described a dual-mode fluorescent ^18^F-PET tracer based on the targeting vector PSMA-617 conjugated to fluorescein and an ammonium tetrafluoroborate.^8^ These very recent studies underline, that zwitterionization is an attractive concept in the field of tumor targeting. However, a systematic evaluation of zwitterionization is currently lacking. Such a systematic study would however be highly valuable as it is likely, that the positioning and the number of zwitterionic groups incorporated into targeted reagents have a strong influence on target binding, cellular uptake and biodistribution of the drug. For PSMA-targeted fluorophores, for example, the linker structure has been shown to affect PSMA-binding properties, cellular uptake and biodistribution significantly.^13,53^ It is likely, that these factors are target specific and will have to be optimized for each target individually to optimize tumor-to-organ ratios for therapy and background-to-lesion ratios for imaging.

This study reports the synthesis and the *in vitro* and *in vivo* evaluation of a series of zwitterionic fluorescent PSMA ligands. The work was designed to address four key questions regarding the role of zwitterionic modifications in PSMA-ligand design: (i) does the positioning and number of zwitterions in the linker region of targeting vectors and effector domains affect PSMA binding; (ii) how do zwitterions influence tumor uptake; (iii) is the site of zwitterionic modification critical for efficacy, and (iv) do zwitterions reduce off-target binding and retention in particularly relevant organs such as head and neck glands and kidneys?

## Results and Discussion

The design of target structures for this study was based on urea derivatives of the PSMA-binding motif of PSMA-617 conjugated to a fluorescent dye. The latter permitted tracking of all compounds with high sensitivity and spatial resolution in cells and *in vivo*. The choice of zwitterionic group, its positioning and the number used for decoration might influence the performance of PSMA-ligands for tumor targeting. Sulfobetaines were selected as zwitterionic groups due to their high stability, accessibility and tendency toward high hydration. The latter property led to efficient antiadhesive properties for proteins and other biomolecules in numerous examples in material science.^48^ Target structures contained different numbers of sulfobetaines either in the linker region or the attached dye. These variations were designed to probe the impact of number and position of zwitterionic groups in PSMA-ligands and to derive guidelines for their rational placement in other PSMA-ligands. In particular, they address whether the position of the zwitterionic group influences PSMA binding affinity and specificity. The fluorescent dyes were selected according to their optical properties, enabling both *in vitro* analysis by cell imaging and *in vivo* evaluation by cryo-fluorescence tomography (CFT). Sulfo-Cy5 (Cy5) was chosen as a water-soluble far-red absorbing dye with one (delocalized) positive charge plus two negatively charged sulfonate groups, while the NIR-dye ZW800−1 **11** was selected due to its excellent optical properties and the ability to probe the effect of two zwitterionic groups in the effector molecule (plus one delocalized positive charge).^38^

The synthesis of all conjugates is shown in Scheme 1. It starts with the solid-phase synthesis of the urea targeting vector on 2-chlorotrityl resin (2-CTC) following a Fmoc solid-phase peptide synthesis protocol. For the synthesis of Cy5−617 **8**, the targeting entity was cleaved from the resin under mild acidic conditions, deprotected in a 1:1 mixture of CH_2_Cl_2_/TFA and subsequently coupled with sulfo-Cy5 NHS ester in anhydrous DMF with DIPEA (Scheme 1). Purification via reversed-phase flash chromatography gave Cy5−617 **8** in 52% yield.

For the linker-modified derivatives, the targeting vector was coupled with Fmoc-l-Aha−OH to introduce an azido-side chain, permitting late-stage conjugation to zwitterions via copper-catalyzed azide−alkyne cycloaddition (CuAAC). The resulting intermediates **2** were cleaved and deprotected to give azides **5**. These were coupled with sulfo-Cy5 NHS ester **4** as described above. Final modification by CuAAC with sulfobetaine alkyne **6** was performed in DMF/H_2_O with CuI and sodium ascorbate at 55 °C. Reaction progress was monitored by HPLC-MS confirming efficient conversion of both compounds to Cy5-ZW-617 **9** and Cy5-ZW_2_-617 **10**. Chromatographic purification of the zwitterionic conjugates can be challenging due to their high polarity and corresponding low retention on RP columns. It is therefore favorable to introduce the sulfobetaines in the last synthetic step. An alternative approach via CuAAC of **2** to **3** and subsequent dye coupling turned out to be not successful. The zwitterionic intermediates **7** were hard to purify and had low reactivity toward the NHS ester **4**. This low reactivity might be due to the kosmotropic effect of the zwitterions leading to shielding of the primary amine in **7.**

Synthesis of the dye conjugate ZW800−617 **13** was not feasible in DMF due to the poor solubility of ZW800−1 **11**. Instead, the reaction was performed in anhydrous DMSO, which dissolved all reactants. Coupling of **12** with HATU and DIPEA proceeded cleanly to the desired product ZW800−617 **13.**

**Scheme 1:**
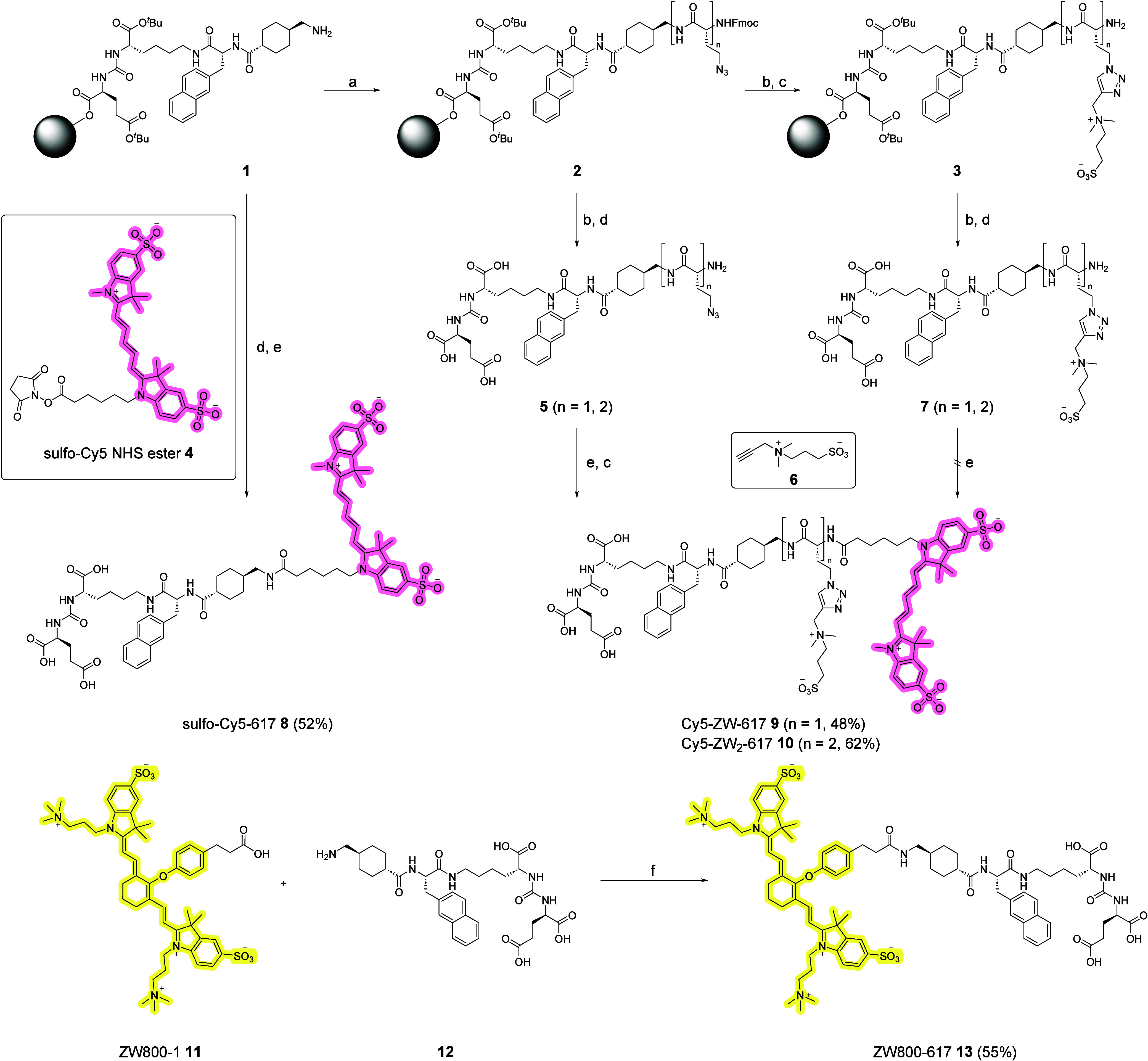
Synthesis of Cy5-617 **8**, Cy5-ZW-617 **9**, Cy5-ZW_2_-617 **10** and ZW800-617 **13** ^a^

a (a) Fmoc-l-Aha−OH, HATU, DIPEA, DMF, 1 h (b) 30% piperidine in DMF (c) sulfobetaine alkyne **6**, CuI, sodium l-ascorbate, DMF/H_2_O 4:1 (d) 2% TFA in CH_2_Cl_2_ *then* TFA/CH_2_Cl_2_ 1:1 (e) sulfo-Cy5-NHS ester **4**, DIPEA, DMF, rt, 18 h (f) HATU, DIPEA, DMSO, rt, 1 h. The chromophores of Cy5 and ZW800-1 are shown in the same colour used for visualization of fluorescence microscopy images in Figures 2 and 3

The impact of the zwitterionic groups on hydrophilicity can be derived from the comparison of the retention times of the compounds measured on a C18 HPLC column (Figure 1A). The trend reveals an increase of hydrophilicity with an increasing number of zwitterionic motifs. Two zwitterionic groups attached to the dye increased the hydrophilicity of the compounds particularly strong. In addition, log*D*_7.4_ values were measured for all dye conjugates (Figure 1B). The log*D*_7.4_ values ranged from − 1.14 to − 1.92 (Figure 1) and are thus higher than those of the radiopharmaceutical [^177^Lu]­Lu-PSMA-617 (log*D*_7.4_ = − 4.4)^55^ and structurally related fluorescent probes based on other urea targeting vectors.^12^ The zwitterionic derivative **13** has a significantly increased hydrophilicity compared to the other compounds investigated here, reflected by the lowest log*D*_7.4_ of − 1.92. This might reflect the larger spacing of negative and positive charges in the zwitterionic dye compared to the sulfobetaines in the side chain. A larger spacer between the opposite charges in sulfobetaines results in a higher dipole moment and can therefore also lead to higher hydrophilicity.^56,57^

To investigate the influence of zwitterionic modifications on serum protein interactions with targeted PSMA ligands, binding to human serum albumin (HSA) was evaluated (Figure 1C). Relative binding affinities were determined for all dye conjugates under the same assay conditions. The Cy5 conjugate **8** showed 60.3% binding to HSA whereas the introduction of a single sulfobetaine in the linker reduced HSA-binding to 26.9%. A second sulfobetaine further decreased HSA-binding to 15.3%. The zwitterionic dye conjugate ZW800−617 **13** led also to a similar reduction in HSA-binding to 16.3%, comparable to **10** with two sulfobetaines in the linker region of the molecule.

The data reveal a relationship between increasing “zwitterionicity” and enhanced hydrophilicity according to decreased retention times measured on a RP-HPLC. log*D*_7.4_ values are similar for compounds **8**-**10** and only compound **13** has a significantly lower log*D*_7.4_ value. HSA-binding is significantly reduced with increasing zwitterionicity. The positioning of the zwitterions had an influence on hydrophilicity, but almost no influence on HSA-binding.

The binding affinities of all PSMA ligands to recombinant human PSMA (rhPSMA) were determined using an enzymatic assay established by Kozikowski *et al.*.^58^ IC_50_ values were measured for all four dye conjugates and PSMA-617 as a reference (Figure 1D and Table S3). The IC_50_ values for all new compounds **8**-**13** ranged from 4.08 ± 2.96 nm to 6.26 ± 3.66 nm. The observed values are all very similar indicating that neither the modification with zwitterions in the dye nor in the linker affected PSMA binding of the targeting urea motif. The reference compound PSMA-617, gave a similar IC_50_ of 2.41 ± 0.09 nm under the same assay conditions (see Table S3, literature value for comparison: 4.61 ± 1.33 nm).^59^

**Figure 1:**
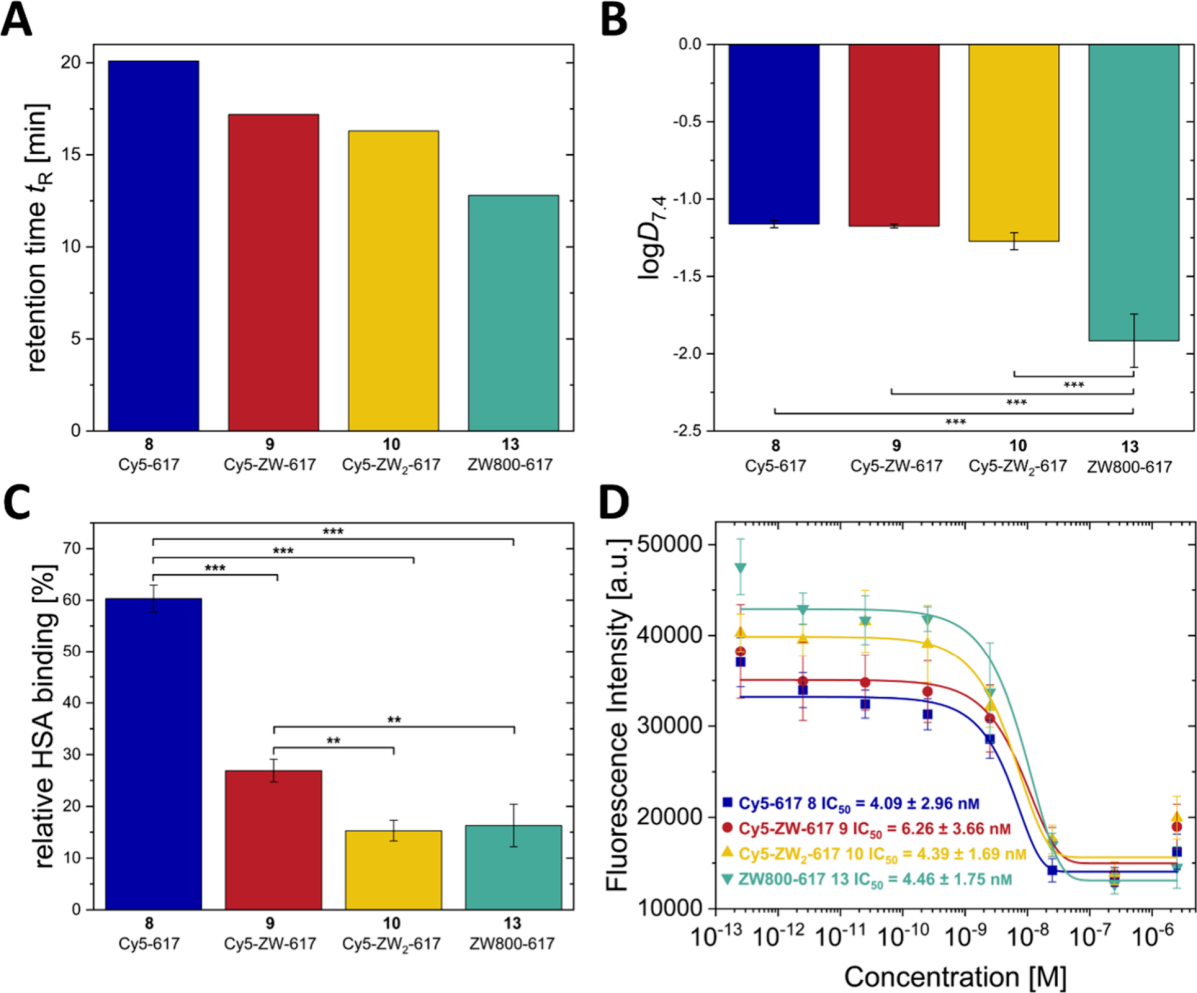
Comparison of (A) retention times *t*_R_ (HPLC, C18), (B) octanol/PBS partition coefficient log*D*_7.4_ ± SD (*n* = 3) (C) relative HSA binding ± SD (*n* = 3) and (D) rhPSMA inhibition constants (IC_50_, *n* = 3) of the Cy5 dye conjugates **8**, **9**, **10** and the ZW800 conjugate **13**. Significant differences are indicated by asterisks (***p < 0.001, **p < 0.05).

In a next step, cell experiments were performed to assess the PSMA-dependent cellular uptake and the subcellular localization of the Cy5 conjugates by widefield fluorescence microscopy (Figure 2). Cell experiments were performed with the PSMA-positive cell lines PC3-PIP and LNCaP, as well as the PSMA-negative PC3 flu cell line to determine uptake specificity. PSMA specificity was further verified by blocking experiments using excess of the strong PSMA-binder 2-(phosphonomethyl)-pentanedioic acid (2-PMPA).^60^ Cells were incubated at two different concentrations of 2.5 μm and 10 μm with the PSMA-ligands for 2 h at 37 °C. All PSMA-ligands tested were efficiently internalized by the PSMA-positive PC3-PIP and LNCaP cells and showed no uptake in PC3 flu cells (for the complete set of negative control experiments see Figure S1). The specific binding to PSMA was furthermore demonstrated by blocking of PSMA-binding with 2-PMPA. The data show, that all dye conjugates bind specifically to PSMA-positive cells and are efficiently internalized. Notably, introduction of zwitterionic sulfobetaine moieties in the linker domain of compounds **9** and **10**, and thus near to the targeting urea motif, did also not compromise cellular uptake significantly. The NIR fluorophore **13** cannot be imaged using the setup employed due to its far-red-shifted excitation. This compound was therefore evaluated separately (*vide infra*).

**Figure 2:**
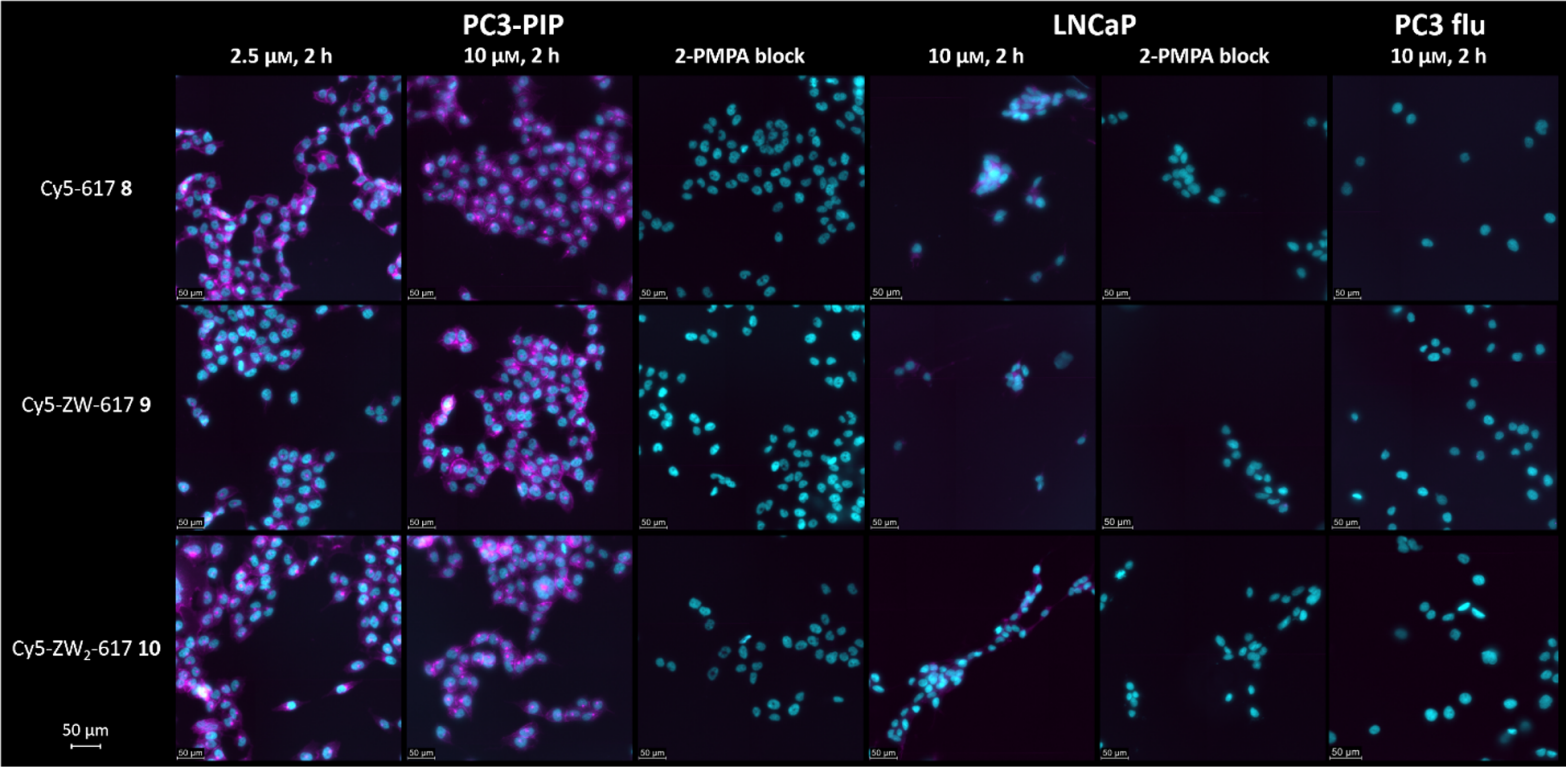
Widefield fluorescence microscopy images of PC3-PIP, LNCaP and PC3 flu cells after incubation with Cy5-conjugates **8**, **9** and **10** (2.5 μm or 10 μm, 37 °C, 2 h) with or without blocking (100-fold excess 2-PMPA). Nuclei were stained with DAPI (cyan). The Cy5 signal is shown in magenta. λ_exc_ (DAPI) = 395 nm; λ_exc_ (Cy5) = 640 nm. Scale bar = 50 μm.

The subcellular distribution of all PSMA-conjugates, was analyzed by confocal laser scanning microscopy with high spatial resolution (Figure 3). All Cy5 conjugates **8**-**10** (Figure 3A) and ZW800−617 **13** (Figure 3B) led to good cellular uptake in PSMA-positive cell lines and a similar subcellular localization. The highest fluorescence intensity was observed in perinuclear compartments. Further control experiments (incubation of PSMA-negative PC3-flu cells with all dye conjugates) are not depicted in Figure 3 but are available in the SI (Figure S3). They confirm the PSMA-specific binding of all dye conjugates including ZW800−617 **13**. These results indicate that zwitterionic moieties can be introduced in the linker domain and in the effector domain of PSMA-ligands without a significant impact on PSMA-specific cellular uptake. In addition, the number and position of zwitterionic groups does not seem to have an impact on the subcellular localization of the compounds.

**Figure 3:**
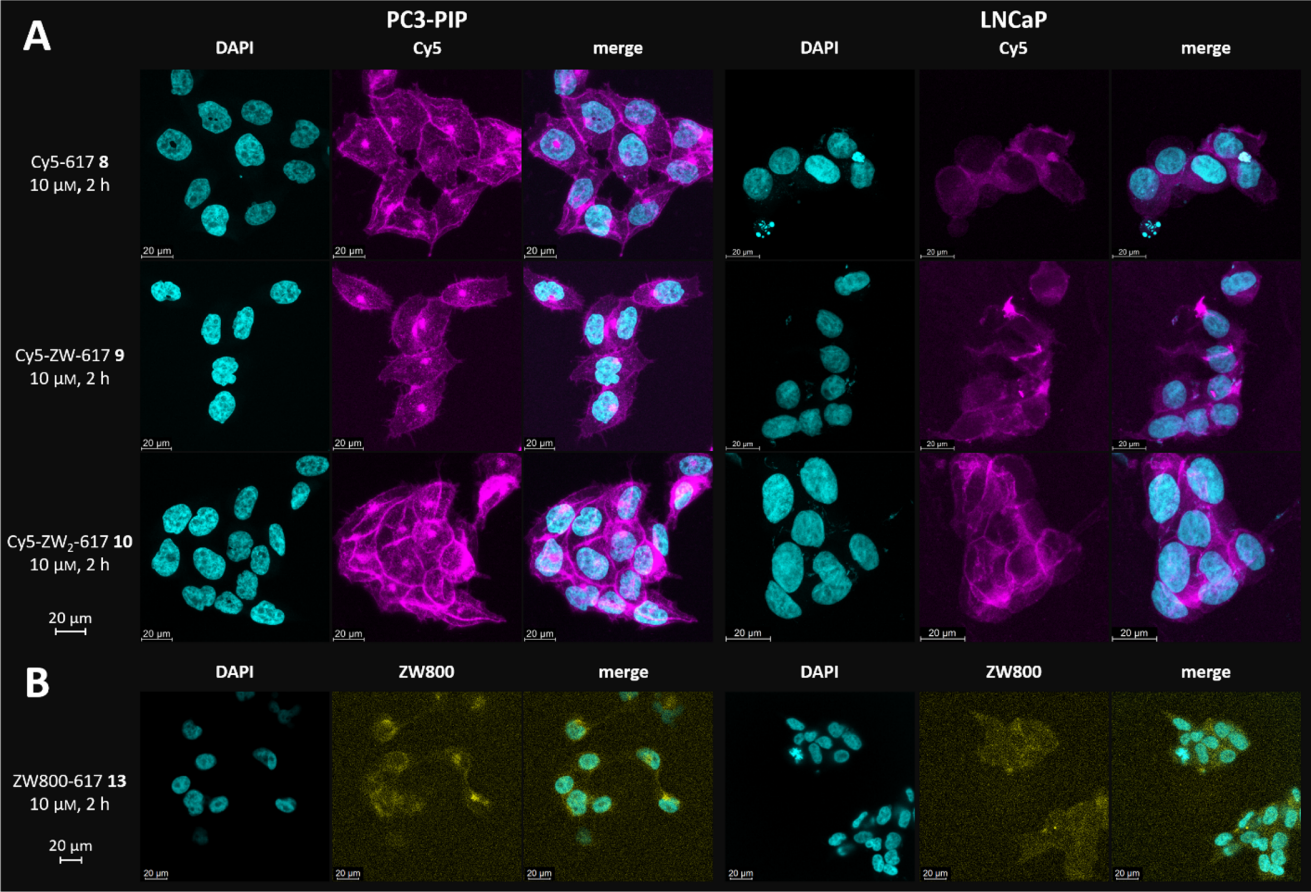
Representative confocal microscopy images of (A) PC3-PIP and LNCaP cells after incubation with Cy5 conjugates **8**, **9** and **10** (10 μm, 2 h, 37 °C) and (B) ZW800−617 **13** (10 μm, 2 h, 37 °C). Nuclei were stained with DAPI (cyan). The Cy5 signal is shown in magenta and the ZW800 signal in yellow. Images are shown as maximum intensity projections of z-stacks acquired with a 63x oil immersion objective. λ_exc_ (DAPI) = 405 nm; λ_exc_ (Cy5) = 653 nm, λ_exc_ (ZW800) = 770 nm. Scale bar = 20 μm.

The impact of “zwitterionization” on the *in vivo* behavior of the dye conjugates was evaluated in mice using CFT. Unlike conventional *in vivo* fluorescence imaging, CFT is a three-dimensional (3D) histological method that provides both high spatial resolution and sensitivity.^61^ Following euthanasia, the animals were embedded in a frozen block and serially sectioned *via* removing 45 μm-thin slices. After removing each slice the sectioned block was imaged under white light and fluorescence excitation. The resulting data sets were computationally reconstructed to visualize anatomical structures and precisely localize the fluorescent probes.

Quantitative high-resolution biodistribution analyses were performed by CFT with a time delay of 4 h postinjection (p.i.) of the dye conjugates (20 nmol) in MMTV-PyMT (spontaneous, syngeneic, breast cancer model) mice (n = 2 per compound). These mice were used as a control group to assess the effect of zwitterionic modification on pharmacokinetics and organ distribution in the absence of a PSMA-positive tumor (Figure 4). The PSMA-617 analogue Cy5−617 **8** showed a relatively slow elimination and was detected in various organs 4 h p.i.. Particularly high retention was observed in the kidneys, lacrimal ducts, and masseter muscles. Compound **8** is expected to bear the least hydrated sulfobetaine among all compounds tested, because the positive charge of the indolium nitrogen is delocalized, whereas all other compounds contained isolated ammonium ions. This property can explain the high off-target tissue retention of **8**. In contrast, the analogue Cy5-ZW-617 **9** with one sulfobetaine in the linker domain, led to a significantly reduced off-target tissue retention. A residual body dose (RBD) of 1.9 nmol corresponding to approximately 10% of the initially injected dose was detected 4 h p.i. for compound **8**. In comparison, Cy5-ZW-617 **9** led to a reduced RBD of 0.8 nmol (58% reduction compared to **8**). Introduction of a second sulfobetaine moiety in the linker further reduced the RBD to 0.25 nmol for Cy5-ZW_2_-617 **10**, equivalent to 87% RBD reduction compared to **8**. The maximum intensity images depicted in Figure 4 reveal very low uptake of **9** and **10** in the lacrimal ducts and salivary glands, only a minimal renal signal and almost complete elimination apart from residual signal in the bladder and urinary tract. The quantitative evaluation revealed a reduction of 81% in renal retention and 98% in the combined head and neck region (including salivary, lacrimal ducts, parotid glands, and masseter muscles) for compound **10** (two sulfobetaines) compared to the reference compound **8.**

For the PSMA-ligand containing the zwitterionic dye ZW800−1 (ZW800−617 **13**) a similar biodistribution profile was observed. However, **13** led to a slightly higher residual signal in the kidneys and a higher RBD of 1.0 nmol compared to the linker-modified derivative **10**. These results demonstrate that both zwitterionic groups in the linker domain and in the dye substantially reduce off-target organ retention. However, the largest effect was observed for compound **10** with two sulfobetaines in the linker domain.

**Figure 4:**
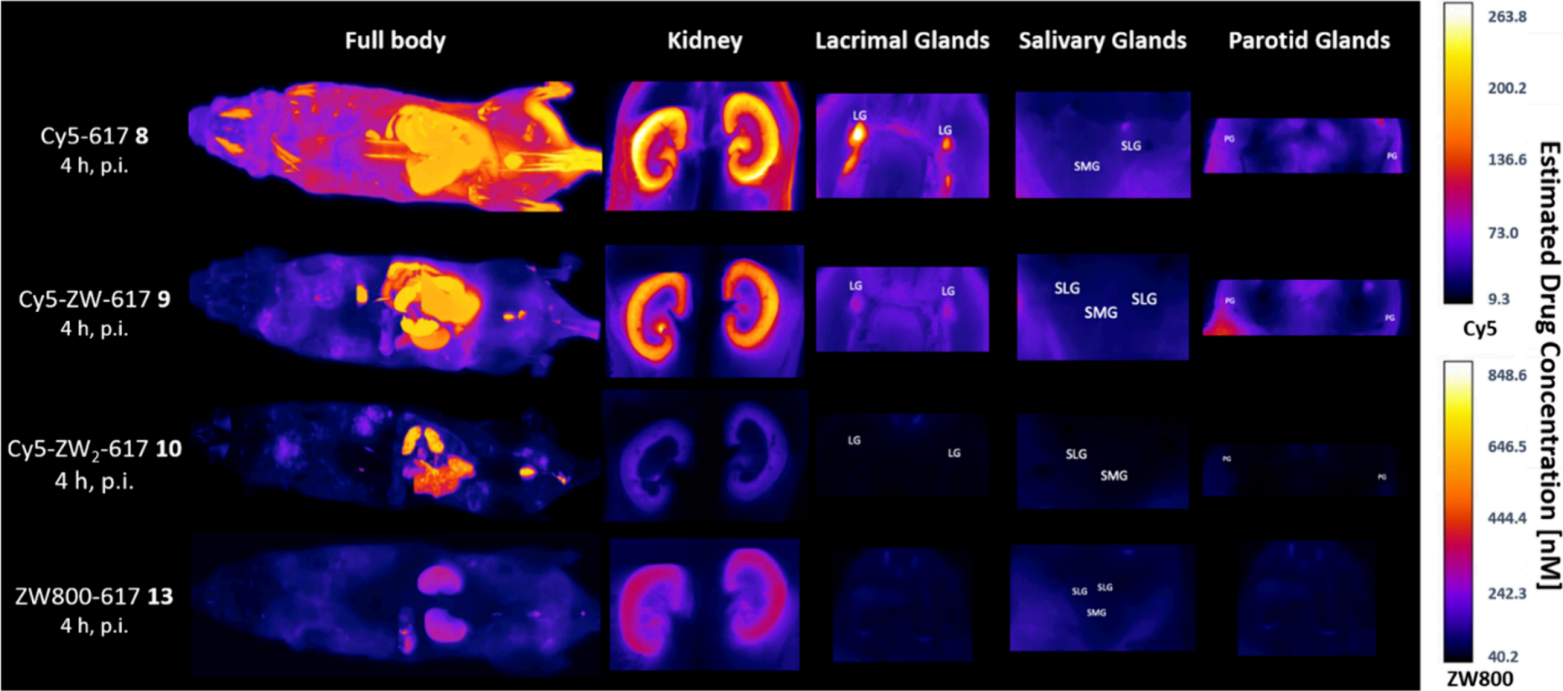
Full body cryo-fluorescence tomography images of PSMA-negative tumor-bearing MMTV-PyMT mice obtained 4 h p.i. of 20 nmol dose of Cy5 conjugates **8**, **9**, **10** and ZW800 conjugate **13**. Depicted maximum intensity images from kidneys, lacrimal, salivary and parotid glands. Selected organs are marked with the following abbreviations: LG = lacrimal glands; SLG = salivary glands; SMG = submandibular glands, PG = parotid glands. Images were captured at 45 μm/pixel resolution with the following set ups: Cy5 channel = 640 nm excitation laser and 680/13 nm emission filter; ZW800 channel = 780 nm excitation laser and 840/70 nm emission filter.

The impact of zwitterionization on *in vivo* tumor uptake and PSMA-specificty was evaluated in tumor xenograft mice bearing a xenograft derived from PC3-PIP (PSMA+) and PC3 flu (PSMA−) subcutaneous injected cells in opposite flanks of the same mouse. The resulting CFT images 4 h p.i. are shown in Figure 5. It should be noted that the intensity maps have been calibrated to the highest observed intensity and are therefore different for each type of fluorophore. The apparent signal in the thorax region of the animal treated with compound **10** is actually fluorescent urine adsorbed to fur and is not internal to the body. The data revealed a specific uptake of all compounds in the PC3-PIP tumors. The uptake for all Cy5-conjugates **8**-**10** and the ZW800-conjugate **13** in the PSMA-positive PC3-PIP tumor was high, whereas only low uptake was detected for the PSMA-negative PC3 flu tumor. The PSMA-specific uptake of all fluorophores was thus confirmed *in vivo.*

**Figure 5:**
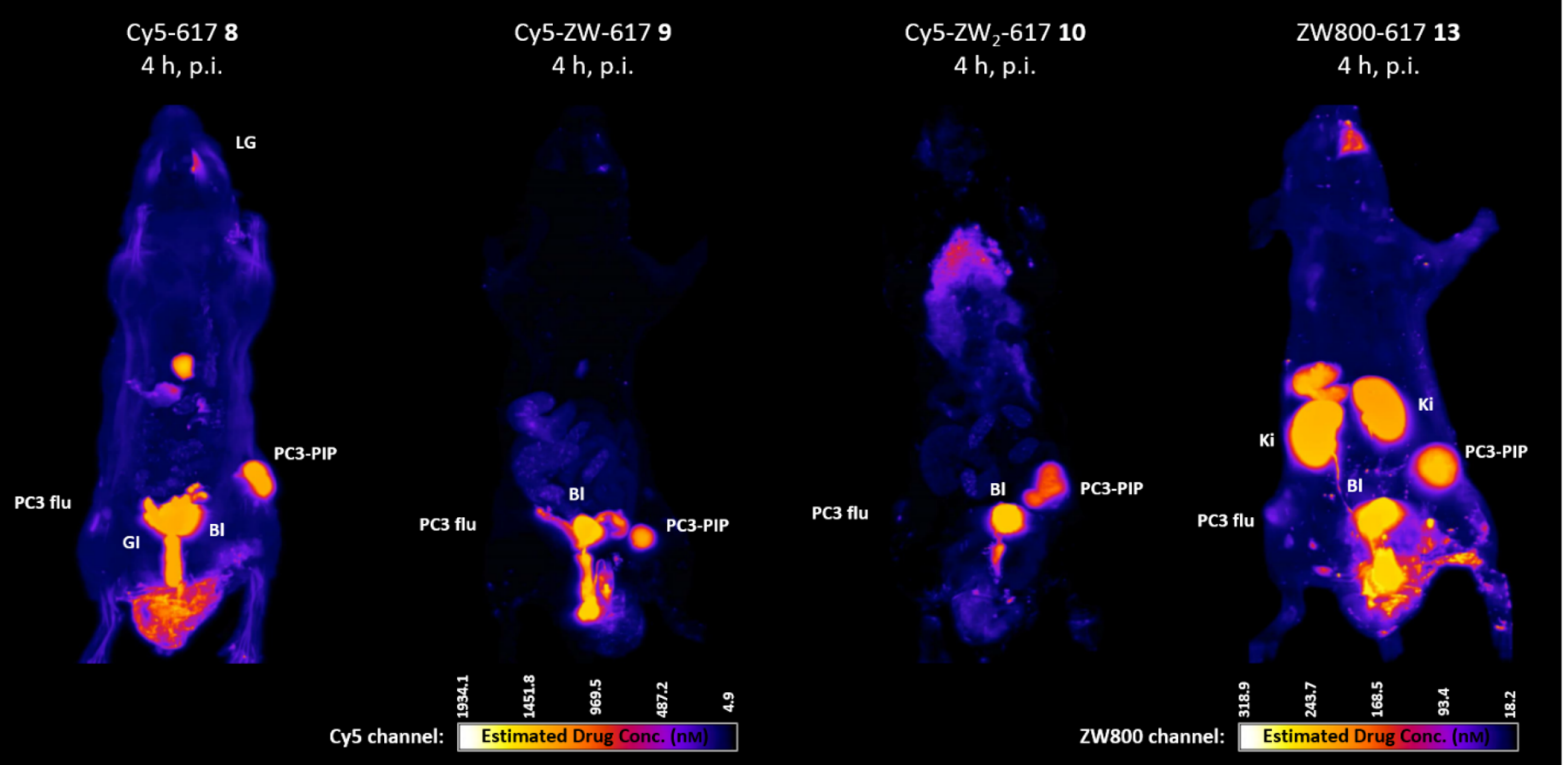
Full body cryo-fluorescence tomography images (maximum intensity projection) of PC3-PIP and PC3 flu tumor-bearing xenograft mice obtained 4 h p.i. of 20 nmol dose of Cy5 conjugates **8**, **9**, **10** and ZW800 conjugate **13**. Selected organs are marked with the following abbreviations: PC3-PIP = PSMA-positive tumor xenograft; PC3 flu = PSMA-negative tumor xenograft; LG = lacrimal glands; Bl = bladder; GI = gastrointestinal tract, *K*_i_ = kidney. Images were captured at 45 μm/pixel resolution with the following set ups: Cy5 channel = 640 nm excitation laser and 680/13 nm emission filter; ZW800 channel = 780 nm excitation laser and 840/70 nm emission filter.

The biodistribution of the PSMA-targeted fluorophores was evaluated for selected compartments by quantitative analysis of the CFT measurements. The quantitative data confirmed the high uptake of all compounds tested in the PC3-PIP tumor. However, the derivatives bearing zwitterionic groups showed a slightly decreased uptake compared to the reference compound **8**, which is probably due to their fast pharmacokinetic and thus shorter contact time with the target. The PC3-PIP tumor uptake of compound **9**, which bears one zwitterionic group in the linker domain, was reduced by approximately − 40% compared with the reference compound Cy5−617 **8**. Incorporation of a second sulfobetaine resulted in a further, though less pronounced, reduction in PC3-PIP tumor uptake (see Figure 6A). ZW800−617 **13** exhibited the lowest PC3-PIP tumor uptake in this series, with a reduction of − 87.5% compared to Cy5−617 **8**. These reduced uptake values must be interpreted in the context of compound retention in off-target compartments and uptake in the PC3 flu tumor. Figure 6A shows the uptake in the two tumor xenografts, the head and neck region, kidneys and muscle. The introduction of zwitterionic moieties into the fluorophores (compounds **9**, **10** and **13**) led to a reduction in uptake in all compartments. Notably, the uptake in the head and neck region was reduced by 15-fold for Cy5-ZW-617 **9**, 28-fold for Cy5-ZW_2_-617 **10** and 9-fold ZW800−617 **13** compared with the reference compound Cy5−617 **8**. A comparable trend was observed for the kidneys and muscle.

To evaluate imaging quality, tumor-to-organ ratios were determined to describe the tumor-to-background relationship. In addition, PC3-PIP tumor to PC3 flu tumor ratios were calculated to reveal differences in PSMA-specific tumor uptake independent of pharmacokinetic effects (see Figure 6B). The tumor-to-organ ratios revealed a clear and consistent trend. The linker-modified derivatives **9** and **10** led to significantly improved tumor-to-muscle ratios (approximately 3-fold (**9**) and 4-fold (**10**) improvement compared to **8**) and an improved tumor selectivity of the same order for both compounds. Particularly notable is the dramatical increase in tumor-to-head and neck region (including salivary and lacrimal glands). Compound **9** led to a 9-fold and compound **10** to a 12-fold improvement in selectivity of this ratio. The numerical values of the ratios can be found in Table S2 in the Supporting Information. The zwitterionic dye conjugate **13**, had a smaller effect on tumor-to-organ ratios, which is due to the lower uptake in the PC3-PIP tumor xenograft. For PC3-PIP tumor to PC3 flu tumor ratios, compound **13** has also only a small effect, which is due to slightly higher uptake of this compound in the PSMA-negative tumor xenograft and the lower uptake in the PC3-PIP tumor xenograft (Figure 6B).

In summary, the introduction of zwitterionic groups led to a reduction in off-target retention resulting in enhanced imaging contrast and superior tumor selectivity particularly for the linker modified compounds **9** and **10** when compared to the reference compound **8.**

**Figure 6:**
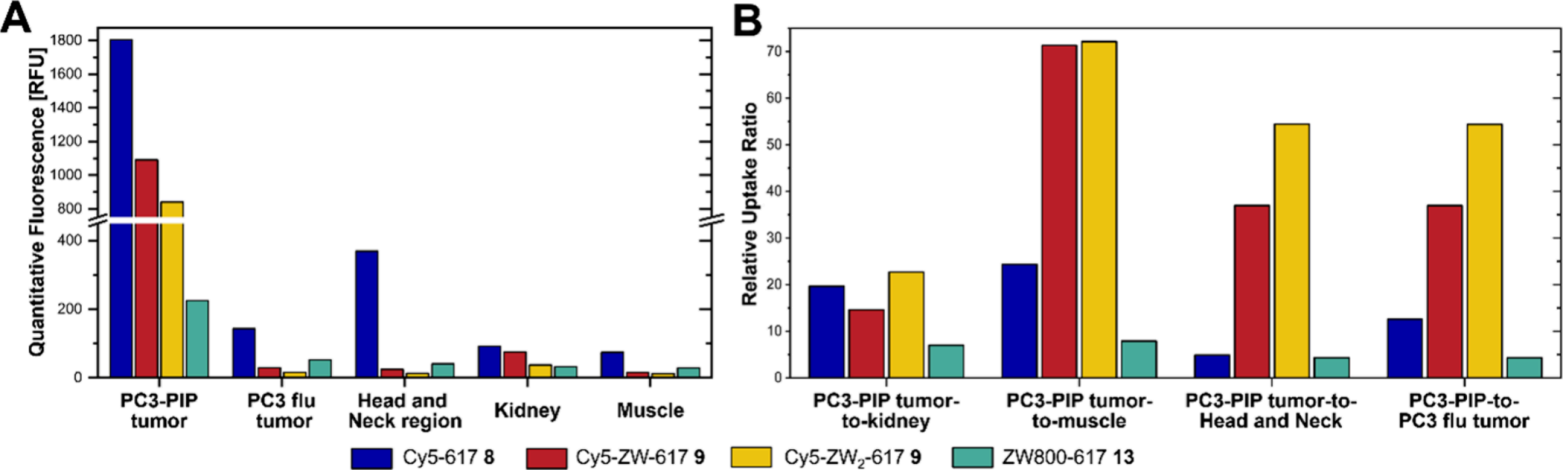
(A) Biodistribution of all dye conjugates in selected compartments obtained by quantification of the CFT fluorescence signal 4 h p.i. of a 20 nmol dose. Quantified compartments include the head and neck region (including salivary, submandibular and parotid glands), the PC3-PIP and PC3 flu tumors, kidneys and muscle. (B) Relative uptake ratios 4 h p.i. illustrating tumor-to-muscle and tumor-to-tumor relationships.

## Conclusion

The presented data demonstrate the influence of zwitterionic groups on the binding properties and biodistribution of fluorescent PSMA ligands. The design of fluorescent probes **8**, **9**, **10** and **13** was based on the clinically successful structural motif of PSMA-617. The introduction of zwitterionic groups into the linker domain between the PSMA-targeting vector and the fluorescent dye is particularly advantageous compared to zwitterionic dyes, although both show dramatic improvements in the tumor-to-background ratio. A major practical advantage of this strategy is its simple synthetic realization using solid-phase peptide synthesis with commercial building blocks and their modification using click-chemistry. The approach is therefore easily transferable to other targeting vectors and alternative effector molecules, such as other dyes and/or metal chelators.

Despite the spatial proximity to the PSMA-binding urea motif, the introduction of two zwitterionic groups into compound **10** did not lead to reduced PSMA binding affinity as demonstrated with an enzymatic binding assay and cell experiments. The excellent PSMA-specific binding properties of the PSMA-617 targeting vector was therefore retained. At the same time, binding to serum albumin was almost completely suppressed by the zwitterionic modification. These properties translated into favorable *in vivo* distribution of fluorophores **9**, **10** and **13**. Particularly linker-modified compounds **9** and **10** led to high PSMA-specific tumor uptake and extremely low off-target retention in mice. This advantageous biodistribution may be due to the high hydration of the sulfobetaine groups used, which shields the linker domain and the dye from nonspecific protein binding. This stealth effect, well-known in materials chemistry, is not limited to sulfobetaine groups but could also be achieved with other zwitterions in the future in the field of tumor targeting.^62^

It is notable that the placement of zwitterionic modification is important: The introduction of two zwitterionic groups into the linker moiety of PSMA-targeted dye conjugate **10** was highly efficient in improving the biodistribution of the compounds whereas the introduction of two zwitterionic groups into the fluorophore structure of **13** had a smaller effect. These results are consistent with those of other recent studies that also found a positive influence of hydrophilic groups (PEG-linkers) in the linker region between targeting vector and effector for PSMA and FAP-targeting radiopharmaceuticals.^29,47^ However, the comparison of the influence of sulfobetaines in the linker region with that on the dye should be interpreted with caution. At this stage it was not possible to use structurally identical sulfobetaines for modification of the linker and the dye. Instead, the study was limited to commercially available zwitterionic dyes with different carbon spacers for charge separation. The latter can influence the hydrophilicity of zwitterions and might thus also influence their pharmacokinetic properties. This issue will be addressed in future studies with the synthesis of appropriate dyes bearing identical sulfobetaine groups.

The observed increase in PC3-PIP tumor selectivity for the zwitterionic compounds **9** and **10** is remarkable. We attribute the low retention of these reagents in off-target compartments to the aforementioned stealth effect of the sulfobetaine groups. In addition, zwitterionic groups in the linker motif (near the PSMA binding urea group) might also lead to the selective targeting of tissue-specific target modifications. It is notable in this context that tissue-specific differences in the glycosylation pattern of PSMA are known,^35,36^ which could lead to reduced retention of charged or zwitterionic PSMA ligands, for example, in the salivary glands or kidneys. Indeed, the slight reduction in tumor uptake might not only be due to the faster pharmacokinetic of the zwitterionic compounds but might also reflect variation in PSMA glycosylation at the cellular level within a particular tumor mass.

The approach demonstrated here is modular. Although the findings reported here were obtained with dye conjugates, it can be assumed that the modification of other PSMA ligands (e.g., corresponding radiopharmaceuticals) with zwitterionic groups has a similarly positive effect on their biodistribution. Corresponding studies are currently underway.

## Experimental Section

### General

The solid-phase bound PSMA targeting entity **1** was synthesized using 2-CTC resin support following an established protocol.^63^ The sulfobetaine alkyne **6** was synthesized according a previously published protocol by Niu et al.^64^ The purity of all compounds was determined to be >95% according to HPLC (UV detection at 254 nm).

All commercially available reagents and starting materials were purchased from Sigma-Aldrich, Iris Biotech, Alfa Aesar or TCI and were used without further purification. Sulfo-Cy5 NHS ester **4** and ZW800−1 **11** were purchased from Lumiprobe GmbH. 2-(Phosphonomethyl)-pentanedioic acid (2-PMPA) was purchased from Cayman Chemical. PSMA-617 was purchased from BLDpharm. Solid phase synthesis was performed using 2 mL polypropylene syringes equipped with filter frits (Carl Roth). Solvents in HPLC grade were purchased from VWR chemicals. Water was purified using an ELGA PURELAB Classic UV water system. The reaction progress was monitored by cleaving a small portion of resin in CH_2_Cl_2_ with 5% TFA for 5 min, followed by filtration and analysis of the supernatant *via* HPLC-MS. UV−vis spectroscopy was performed on a Thermo Fisher Genesys 10-S UV−vis spectrophotometer.

Medium pressure liquid chromatography was performed on automated systems using prepacked cartridges with 15 μm C18AQ silica (Interchim). Reversed phase flash chromatography was carried out on an Interchim PuriFlash 430 system or a Büchi Pure C-850 Flash/Prep system with MeCN/H_2_O containing 0.1% formic acid as the mobile phase.

Analytical HPLC-MS was performed on an Agilent HPLC system 1260 Infinity II with a Macherey-Nagel NUCLEODUR C18 Gravity-SB column (3 μm, 100 × 2 mm) coupled to a Bruker amaZon SL ion trap mass spectrometer with an ESI source. The parameters of the HPLC-method are given in Table 1.

**Table 1:** HPLC Conditions

Time	% H_2_O (0.1% FA)	% MeCN (0.1% FA)	Flow rate (mL/min)
0−2 min	98	2	0.2
2−20 min	2	98	0.2
20−23 min	2	98	0.2
23−25 min	98	2	0.2
25−30 min	98	2	0.2

### Statistics

A one-way ANOVA followed by Tukey’s multiple-comparison test was performed to analyze statistical significance. P values <0.05 were considered statistically significant. Sample sizes (n) are provided in the respective figure captions.

### Determination of log*D* (Octanol_,_ PBS, pH 7.4) Values

The log*D*_7.4_ values were determined using a shake-flask method followed by quantification via HPLC. Analyses were performed on the above-mentioned HPLC system with a DAD detector, ensuring that injected amounts and resulting peaks were within the detectors linear range. Octanol was saturated with water by vortexing a 1:1 mixture of *n*-octanol and PBS buffer (pH 7.4, Gibco) for 2 min, followed by phase separation via centrifugation (6000 rcf, 30 min). Equal amounts of each dye conjugate were dissolved in 2 mL aqueous PBS buffer (pH 7.4) and 1 mL of the solution was mixed with 1 mL of water saturated octanol. Samples were vortexed and then shaken at room temperature for 2 h before complete phase separation by centrifugation (6000 rcf, 30 min). The initial aqueous solution (reference) and the octanol phase were then analyzed by HPLC. The peak areas were determined using the peak integration function of Compass HyStar and the log*D*_7.4_ values were calculated based on the decrease in peak area (636 nm) in the water phase.

### Relative Binding to Human Serum Albumin via an Ultrafiltration Assay

The relative albumin-binding affinity of the dye conjugates was assessed using human serum albumin (HSA, PAN Biotech, Premium grade) and Amicon centrifugal ultrafiltration inlets with a molecular weight cutoff at 30 kDa. First the filtration inlets were washed with 400 μL PBS buffer (pH 7.4) via centrifugation and the volume was kept inside the vial. Dye conjugate solutions (100 μm) were prepared in PBS buffer and their concentration was confirmed via UV−vis spectroscopy. Then 374 μL of each dye solution was either substituted with 26 μL PBS buffer (reference) or 26 μL of the HSA solution to derive a final concentration of 200 μm HSA. Both solutions were then incubated at 37 °C for 20 min and then loaded on the ultrafiltration inlets. Separation was performed via centrifugation (17000 rcf, 30 min, 4 °C) and the unbound fraction of dye conjugates were quantified by UV−vis spectroscopy of the filtrate. The filtrate of the reference solution was quantified to determine the unspecific binding on the membrane. For Cy5 the measurements were conducted at 650 nm and for ZW800 at 780 nm.

### Binding Affinity via NAALADase Assay

The *in vitro* PSMA binding affinity was determined enzymatically following an established protocol reported by Kozikowski et al. via a fluorescence-based NAALADase assay.^58^ A 0.4 μg/mL solution of recombinant human PSMA (rhPSMA, Bio-Techne GmbH) and a 40 μm solution of NAAG in assay buffer (50 mm HEPES, 0.1 m NaCl, pH 7.4) were prepared. Following solutions of the dye conjugates **8**-**10**, **13** and PSMA-617 as an internal reference in a concentration regime of 10^−5^ to 10^−12^ m were prepared. 12.5 μL of the compound solutions were mixed with 12.5 μL of the NAAG solution in Nunclon Delta Surface black polystyrene 96-well plates and 25 μL of the rhPSMA solution was added. After 1 h incubation at 37 °C the amount of released glutamate was determined using the Amplex Red glutamic assay kit and the fluorescence was measured with a Tecan Spark plate reader with an excitation wavelength λ_exc_ = 535 nm and detection at λ_det_ = 590 nm.

### Cell Culture

The PSMA-positive cell line LNCaP was obtained from DSMZ (Deutsche Sammlung von Mikroorganismen and Zelllinien #ACC 256). LNCaP cells were cultured in RPMI-1640 cell culture medium (PAN-Biotech) supplemented with 10% fetal bovine serum (Capricorn Scientific), 1% penicillin-streptomycin and 1 mm sodium pyruvate (Gibco) in a humidified incubator at 37 °C under 5% CO_2_. At 80−90% confluency, cells were washed with DPBS buffer (Gibco, without Ca^2+^/Mg^2+^) and incubated with 3 mL 0.25% trypsin/EDTA (Gibco) for 3 min. Following the LNCaP cells were subcultured at a seeding ratio of 1:5. The PSMA-positive PC3-PIP and PSMA-negative PC3 flu cell lines were obtained from Prof. Dr. Udo Schumacher (University Medical Center Hamburg-Eppendorf, PC3-PIP) and DSMZ (#ACC 465, PC3 flu), respectively. Both cell lines were cultured in a 1:1 mixture of RPMI-1640 and Ham’s F-12 cell culture medium (PAN-Biotech) supplemented with 10% fetal bovine serum (Capricorn Scientific), 1% penicillin-streptomycin under the same incubation conditions. At 80−90% confluency the cells were passaged as described above and then seeded in a ratio of 1:12.

### Uptake Assay for Cell Imaging

For microscopy experiments 1 × 10^5^ cells per well were seeded in 8-well chambered coverslips (Thermo Fisher Scientific Nunc Lab-Tek, Permanox plastic) and cultured for 24 h in a humidified incubator (5% CO_2_ at 37 °C). For blocking experiments, the cell culture medium was supplemented with 250 μm 2-PMPA and the cells were incubated for 1 h at 37 °C. The medium was then replaced with fresh culture medium containing either 2.5 μm or 10 μm of the dye conjugates, followed by incubation for 2 h at 37 °C. Cells were washed twice with PBS and fixed in 4% formaldehyde in PBS. After two PBS washes the cells were permeabilized using 0.2% triton in PBS for 10 min. Following two PBS washes, nonspecific binding sites were blocked with 1% BSA in PBS for 30 min. Cells were again washed twice with PBS and then stained with DAPI in PBS (1 μg/mL) for 15 min. The cells were washed twice with PBS and mounted with ProLong Diamond Antifade (Thermo Fisher Scientific) mounting medium, covered with 22 × 50 mm microscope cover glasses (Marienfeld Superior) and sealed with clear colorless nail polish.

### Fluorescence Widefield Microscopy

All fluorescence widefield microscopy data were acquired using a Leica DMi8 inverse widefield microscope with a LED excitation light source, a Leica K8-A2 CMOS camera and a 40x air objective (Leica HC PL FLUOTAR L 40*x*/0.60 DRY). DAPI was excited at λ_exc_ = 395 nm and Cy5 at λ_exc_ = 640 nm. Channels were recorded sequentially and then merged.

### Confocal Microscopy

For imaging the Cy5 conjugates confocal microscopy was performed using a Leica SP8 confocal microscope equipped with a white light laser, 4 hybrid detectors and a 63x oil immersion objective (Leica HC PL APO CS2 63*x*/1.40 OIL). DAPI was excited at λ_exc_ = 405 nm and Cy5 conjugates at λ_exc_ = 653 nm. Z-stacks with a thickness of 10 μm were acquired with a step size of 0.3 μm and processed as maximum intensity projections (MIP). Channels were recorded sequentially and then merged. Image processing was performed using Leica LAS X software or ImageJ.

For imaging the ZW-800 conjugate **13** confocal microscopy was performed using a Leica cryoCLEM Stellaris 8 confocal microscope without the cryostage equipped with a white light laser and four hybrid detectors and a 63x oil immersion objective (Leica HC PL APO 63*x*/1.40 OIL). DAPI was excited at λ_exc_ = 405 nm and ZW800 at λ_exc_ = 770 nm. Channels were recorded sequentially and then merged. Images were acquired in a single z level. Image processing was performed using Leica LAS X software.

### Xenograft Preparation

All animal experiments described in this manuscript were conducted in accordance with the ARRIVE guidelines (https://www.nc3rs.org.uk/arrive-guidelines) and protocols were approved by the Institutional Animal Care and Use Committee (IACUC) of Washington University in St. Louis. The laboratory conducting the experiments is accredited under protocol number 24−0832−02.

Male immunocompetent athymic nude-Fox1nu mice (age 6 wk; Inotiv) were implanted with PSMA-expressing and PSMA-negative PC3 xenografts. The tumor cell lines PC3-PIP and PC3 flu were used, and cultured in standard DMEM media (ATCC). Animals were implanted with 5.0·10^6^ cells per xenograft using a 1:1, DMEM:Matrigel mixture (Corning) in a total volume of 100 μL. Subcutaneous injections for PC3-PIP and PC3 flu were performed on the right and left flanks, respectively, and monitored for volume.

### Handling and Dye Injection

Tumor bearing mice with volumes 100−200 mm^3^ were anesthetized with isoflurane at 2−2.5% in air and subsequently injected retro-orbitally (RO) with each compound of interest. Injection volumes were 100 μL (20 nmol dose of each dye conjugate) using a U-100 insulin syringe. Mice were monitored for recovery from the anesthesia, returned to their cages, and then euthanized by computer-controlled CO_2_ at 4 h postinjection and prepared for freezing.

### Whole Mouse Freezing

Following sacrifice the animals were placed on a prechilled metal pan filled with dry ice and covered with dry ice, in a styrofoam cooler, for 45 min. The euthanized animals were placed belly side down with arms, legs, and tails extended to allow for efficient cooling. Once fully frozen the animals were placed into individual low-density polyethylene bags that had been precooled on dry ice, and stored at −80 °C for later use.

### Cryo-fluorescence Imaging

The frozen mice were embedded coronally in a block of Dark OCT 31 measuring 18 cm × 14 cm × 10 cm designed for the Xerra CFT system (EMIT Imaging, Natick, MA). The first block contained eight mice injected with the Cy5 conjugates, and the second block contained all mice injected with ZW800 conjugates. Into the corresponding block, a set of serial dilution standards were included for both Cy5 and ZW800−1 (concentration ranges of 3.91−1000 nm) so the dye-drug concentration could be estimated from the fluorescence signal.

Following autofocusing and trimming, the CFT imaging process of serial anatomical and fluorescence block-face imaging was initiated. CFT imaging was performed with a 12 mega-pixel camera with an image size of 4,096 × 3,008 pixels, resulting in a 45 μm in-plane pixel size. To maintain isotropic voxels, serial sectioning was performed at 45 μm thickness. The Cy5 signal was detected using excitation at 640 nm and filtered detection at 680 nm (13 nm bandpass). The ZW800−1 signal was detected using excitation at 780 nm and filtered detection at 840 nm (70 nm bandpass). To ensure sensitivity without saturation, five fluorescence images were acquired from 5 to 2500 ms.

During image acquisition, both fluorescent and anatomical images underwent corrections to ensure consistency and accuracy: a flat-field correction was applied to compensate for nonuniform illumination and detector response, a dark-field correction was applied to remove background noise and offset variations in the detector, and warping was performed to correct chromatic aberrations. These corrections align the fluorescence and anatomical images and ensure even illumination across the block, enhancing image quality for visualization and analysis.

The acquisition image contains all subjects in one image, so after processing, each subject was segmented from the full block image for visualization and analysis using XerraRecon (EMIT) and Fiji software packages.^65^ Visualization and analysis was performed using Fiji on the optimal exposure time that maximized the 16-bit data range without saturation, which was 50 ms and 500 ms, for Cy5 and ZW800−1 respectively. Visual outputs included flythrough movies of both fluorescent and anatomical image stacks, along with representative axial slices and region-of-interest (ROI) overlays for both fluorescence channels. ROI analysis was performed on selected regions-of-interest as well as a whole-body region to determine residual total drug concentration at each time point. Using the dilution standard, the conversion of fluorescence signal to nm concentration was performed using 18.7x+69 and 61.7x-2382, for Cy5 and ZW800−1 respectively.

## Supplementary Material





## Notes

Additional CFT and microscopy images and CFT-quantification data; procedures for the synthesis of new compounds; HPLC-MS and UV−vis spectra of new compounds (PDF)
SMILES representing the chemical structure of compounds (CSV)

## ABBREVIATIONS

CFT: Cryo-fluorescence tomography
p.i.: Postinjection
HSA: Human serum albumin
NAAG: *N*-acetylaspartylglutamic acid
PSMA: Prostate-specific membrane antigen
rhPSMA: Recombinant human prostate specific membrane antigen
