# Supplementary material for: Zwitterionic Modification of PSMA Ligands Reduces Off-Target Binding and Tissue Retention
Source: J Med Chem. 2026 Mar 13;69(6):7364–76. doi: 10.1021/acs.jmedchem.5c03849 (PMC13036764; doi:10.1021/acs.jmedchem.5c03849)
Supplement: Supplementary file 1 [file jm5c03849_si_001.pdf]

Supporting Information

Zwitterionic Modification of PSMA Ligands Reduces Off-Target Binding and Tissue Retention

Lennart F. V. Spickschen,[a] *Roland Thünauer,*[b] *Aleksander J. Swierzewski,*[a] *John M. Van Wazer,*[c] *Amanda Fears,*[c] *Matthew D. Silva,*[d] *Daniel L. J. Thorek,*[c] *Elke Oetjen,*[e] *Wolfgang Maison*[*, a]

---

[a] Department of Chemistry, Institute of Pharmacy, University of Hamburg, Bundesstrasse 45, 20146 Hamburg, Germany, E-mail: wolfgang.maison@uni-hamburg.de, [b] Technology Platform Light Microscopy (TPLM), University of Hamburg and Advanced Light and Fluorescence Microscopy (ALFM) Facility, Centre of Structural Systems Biology, Notkestrasse 85, 22607 Hamburg, [c] Department of Radiology, Washington University, School of Medicine, 510 S. Kingshighway Blvd., Saint Louis, MO 63110 (USA), [d] EMIT Imaging, Inc., 12 Michigan Drive, Natick, MA 01760 (USA), [e] Institute of Clinical Pharmacology and Toxicology, University Medical Center Hamburg-Eppendorf, Martinistrasse 52, 20246 Hamburg, Germany

Additional Microscopy, CFT tomography images and CFT quantification data

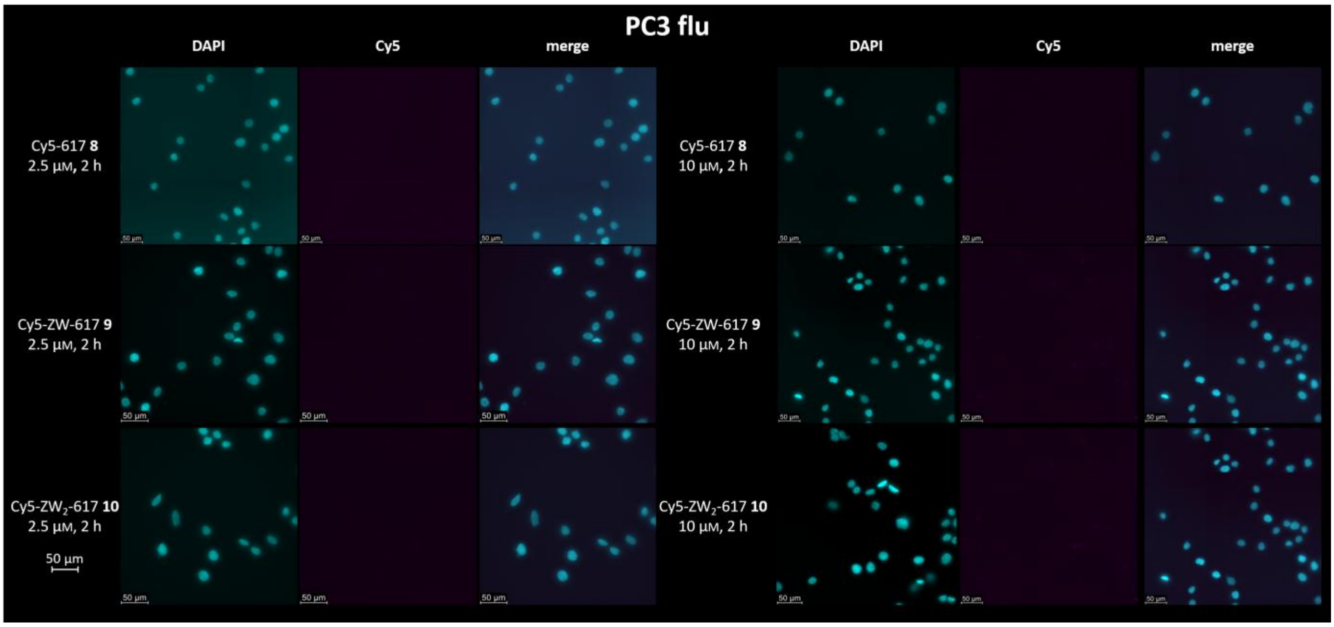

Figure S1: Widefield microscopy images of PC3 flu cells after incubation with **8**, **9** and **10** (2.5 µM or 10 µM, 37 °C, 2 h). Nuclei were stained with DAPI (cyan). The Cy5 signal is shown magenta. λ_{exc} (DAPI) = 395 nm; λ_{exc} (Cy5) = 640 nm. Scale bar = 50 µm.

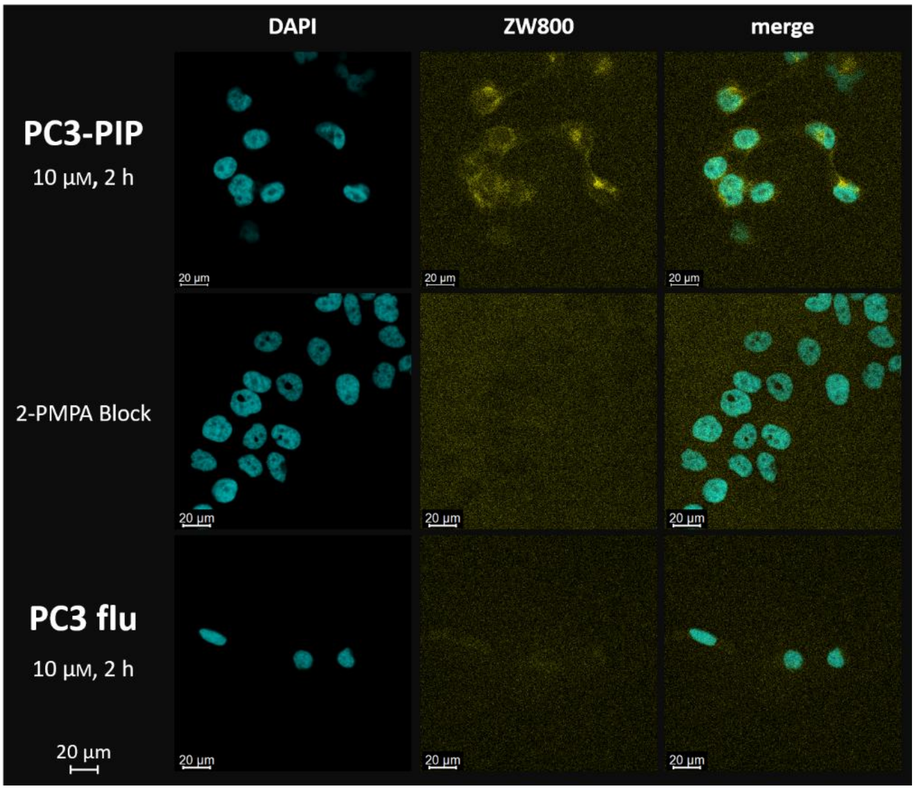

Figure S2: Confocal microscopy images of PC3-PIP and PC3 flu cells after incubation with ZW800-617 **13** (10 µM, 2 h, 37 °C) with or without blocking (2.5 µM **13**, 100-fold excess 2-PMPA, 2 h, 37 °C). Nuclei were stained with DAPI (cyan). The ZW800 signal is shown in yellow. Images are shown as maximum intensity projections of z-stacks acquired with a 63x oil immersion objective. λ_{exc} (DAPI) = 405 nm; λ_{exc} (ZW800) = 770 nm. Scale bar = 20 µm.

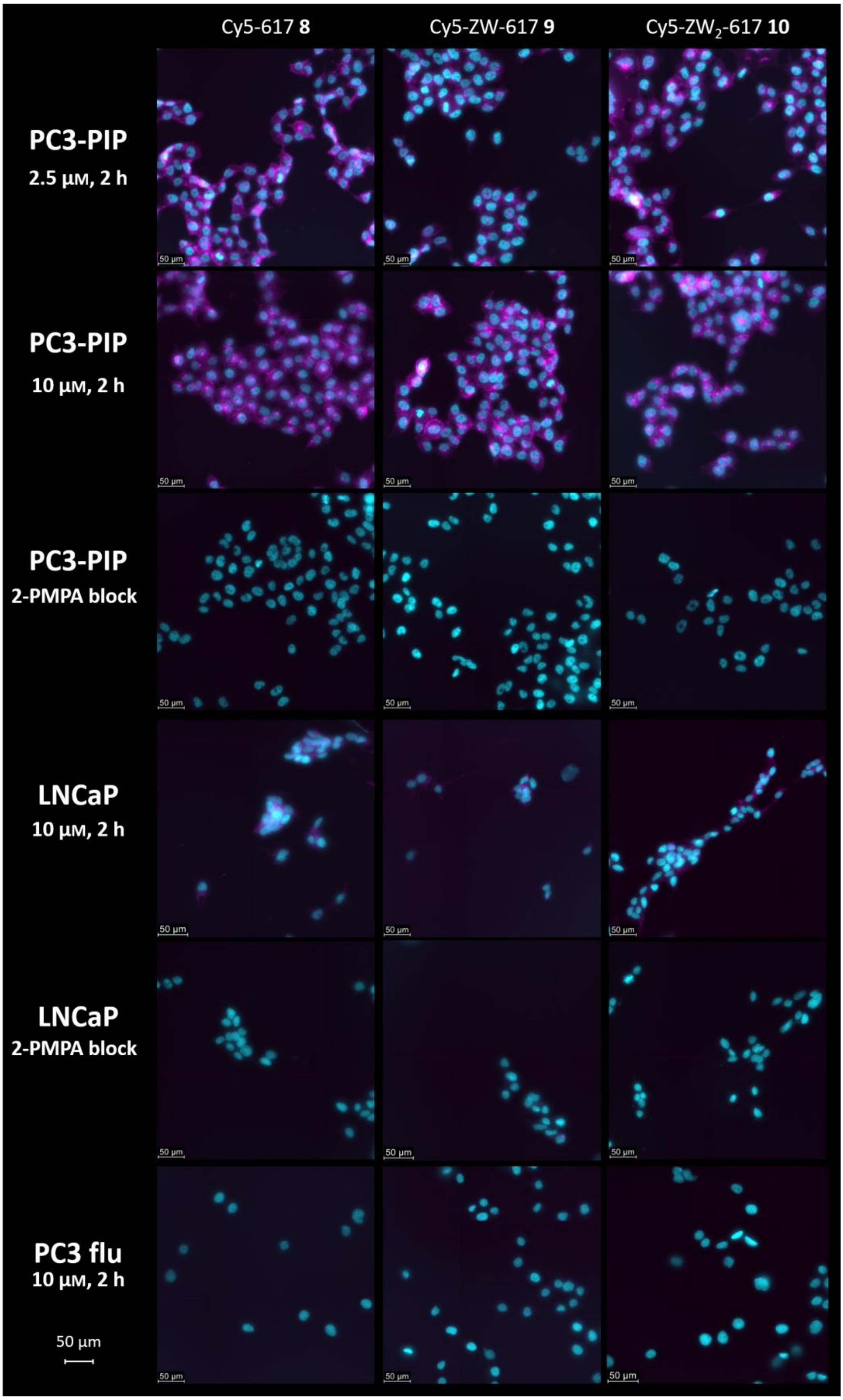

Figure S3: Widefield fluorescence microscopy images of PC3-PIP, LNCaP and PC3 flu cells after incubation with Cy5-conjugates **8**, **9** and **10** (2.5 µM or 10 µM, 37 °C, 2 h) with or without blocking (100-fold excess 2-PMPA). Nuclei were stained with DAPI (cyan). The Cy5 signal is shown magenta. λ_{exc} (DAPI) = 395 nm; λ_{exc} (Cy5) = 640 nm. Scale bar = 50 µm.

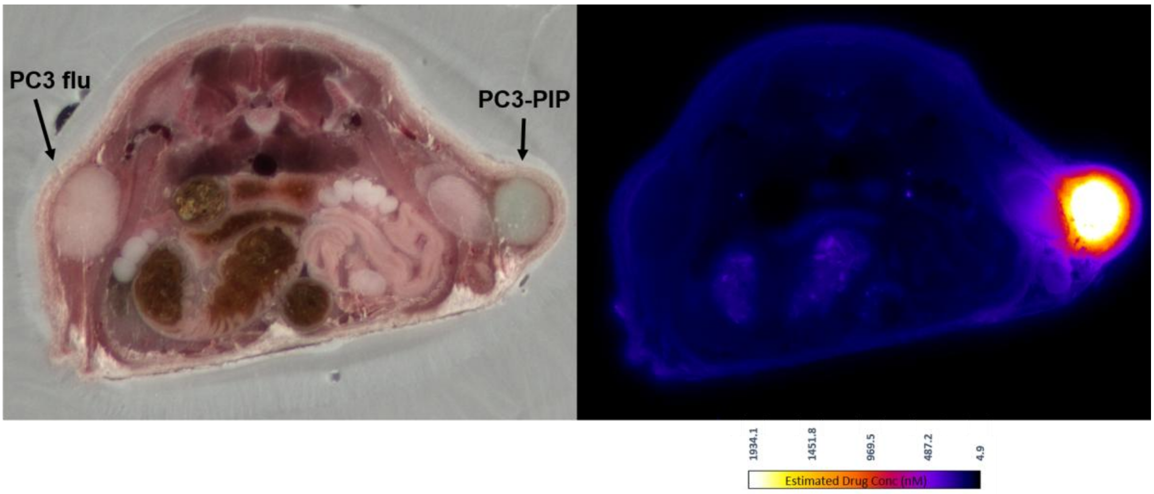

Figure S4: Representative CFT section through the PC3-PIP and PC3 flu tumors showing the fluorescence distribution of Cy5-617 **8**.

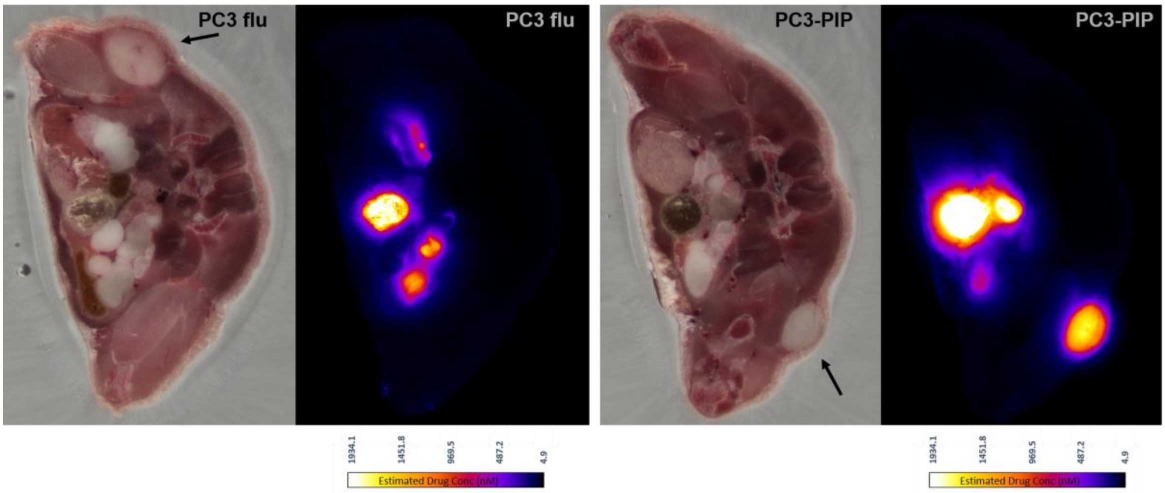

Figure S5: Representative CFT section through the PC3-PIP and PC3 flu tumors showing the fluorescence distribution of Cy5-ZW-617 **9**.

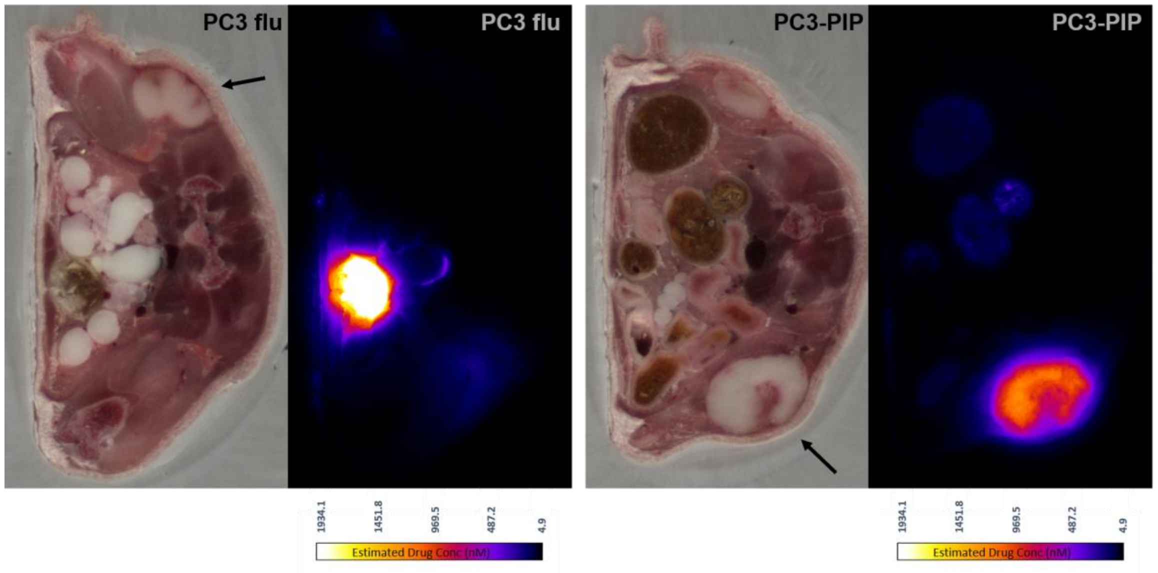

Figure S6: Representative CFT section through the PC3-PIP and PC3 flu tumors showing the fluorescence distribution of Cy5-ZW$_2$-617 **10**.

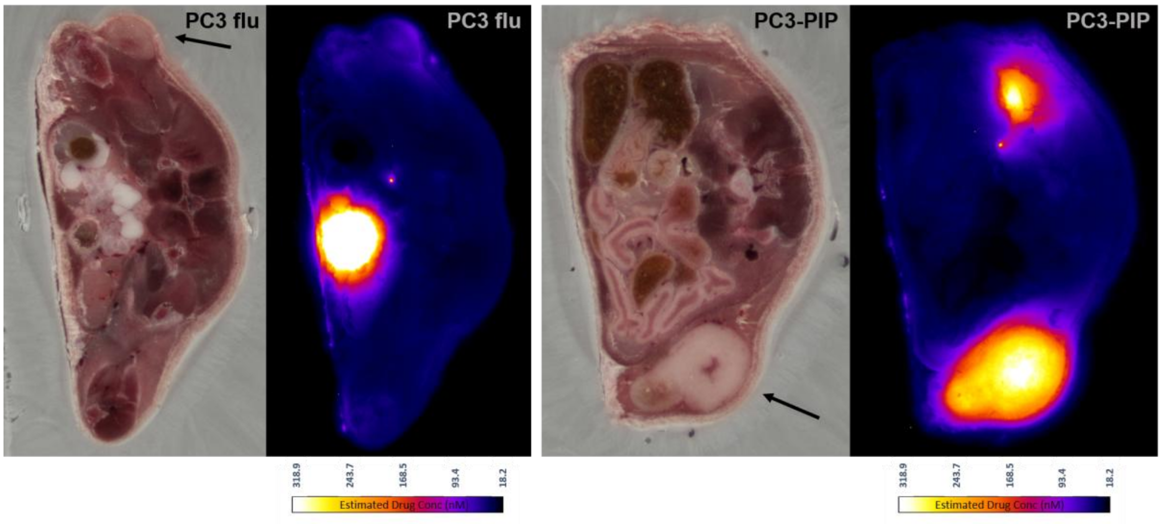

Figure S7: Representative CFT section through the PC3-PIP and PC3 flu tumors showing the fluorescence distribution of ZW800-617 **13**.

Table S1: Quantified CFT fluorescence signals determined for the investigated compartments 4 h post injection of 20 nmol dose.

**Compartment**	**Quantified fluorescence signal [nM]**			
	Cy5-617 **8**	Cy5-ZW-617 **9**	Cy5-ZW_2-617 **10**	ZW800-617 **13**
PC3-PIP tumor	1804.62	1090.31	840.71	224.97
PC3 flu tumor	143.36	29.50	15.46	52.30
Head and Neck	368.81	24.65	13.26	40.71
Kidney	91.74	74.75	37.06	32.13
Muscle	74.07	15.29	11.67	28.55

Table S2: Relative uptake ratios between the investigated compartments 4 h post injection of 20 nmol dose.

**Compartments**	**Calculated relative uptake ratios**			
	Cy5-617 **8**	Cy5-ZW-617 **9**	Cy5-ZW_2-617 **10**	ZW800-617 **13**
PC3-PIP tumor-to-kidney	19.67	14.59	22.69	7.00
PC3-PIP tumor-to-muscle	24.36	71.33	72.14	7.88
PC3-PIP-to-PC3 flu tumor	12.59	36.96	54.38	4.30
PC3-PIP-to-Head and Neck	4.89	44.23	63.40	5.53

Synthesis

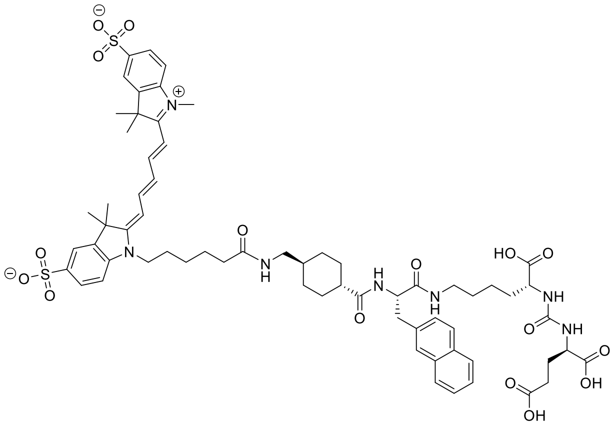

Cy5-617 (8). To a solution of the fully deprotected PSMA-617 targeting entity **1** (10.6 mg, 14.0 µmol, 1.00 eq.) in anhydrous DMF (1 mL) was added a solution of sulfo-Cy5 NHS ester **4** (10 mg, 16.0 µmol, 1.20 eq.) in anhydrous DMF (0.2 mL). The resulting dark blue solution was stirred at room temperature under light exclusion for 19 h. The reaction mixture was diluted with H_2O (7 mL) and lyophilized to afford a blue lyophilizate. The crude product was purified *via* reversed phase flash chromatography (*loaded as solution* in H_2O (1 mL), 15 µm C18AQ silica, Interchim F0004 cartridge, 100% H_2O + 0.1% formic acid holding for 1 cv, then gradient to 100% MeCN + 0.1% formic acid in 15 cv, holding for 3 cv). After lyophilization Cy5-617 **8** (9 mg, 52%) was obtained as a dark blue lyophilizate.

***t*R** (Gravity SB, method 1) = 20.1 min.

HRMS (ESI) *m*/*z* $[M+H]^+$ calcd. for $C_{65}H_{82}N_7O_{16}S_2^{2+}$: 1280.5254, found: 1280.5249.

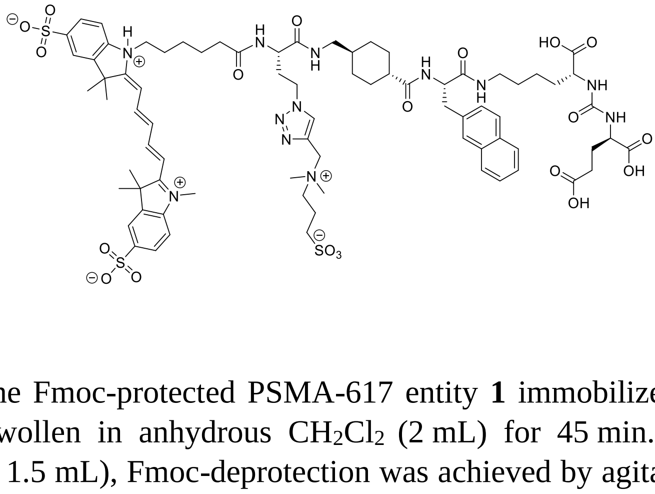

Cy5-ZW-617 (9). The Fmoc-protected PSMA-617 entity **1** immobilized on 2-CTC (50 mg, 0.6 mmol / g) was swollen in anhydrous CH_2Cl_2 (2 mL) for 45 min. After washing with anhydrous DMF (3 x 1.5 mL), Fmoc-deprotection was achieved by agitating the resin in 30% piperidine in DMF (1.5 mL) for 10 min, repeated three times and following the resin was

washed with DMF (3 x 1.5 mL). In parallel Fmoc-L-Aha-OH (33.0 mg, 90.0 µmol, 3.00 eq.) was dissolved in anhydrous DMF (1 mL), DIPEA (41.0 µL, 240 µmol, 8.00 eq.) was added, followed by HATU (34.2 mg, 90 µmol, 3.00 eq.) and the reaction mixture was allowed to activate for 3 min. The resin was agitated in this solution for 1 h and subsequently washed with DMF (3 x 1.5 mL), CH_2Cl_2 (3 x 1.5 mL) and DMF (3 x 1.5 mL). The Fmoc-protected amine was then deprotected under the same conditions as described above, followed by washing with DMF (3 x 1.5 mL) and CH_2Cl_2 (3 x 1.5 mL). Cleavage of the coupling product was carried out by agitating the resin in 2% TFA in CH_2Cl_2 (1.5 mL) twice for 10 min. After washing with CH_2Cl_2 (3 x 1.5 mL) all volatiles were removed *in vacuo*. The crude product was dissolved in CH_2Cl_2 / TFA 1:1 (3 mL) and stirred at room temperature for 16 h. All volatiles were removed *in vacuo* and a portion of the colourless residue (10 mg, 13 µmol, 1.00 eq.) was dissolved in anhydrous DMF (1 mL), followed by addition of DIPEA (17.4 µL, 102 µmol, 8.00 eq.) and sulfo-Cy5 NHS ester **4** (9.5 mg, 13 µmol, 1.00 eq.). The resulting dark blue solution was stirred at room temperature under light exclusion for 16 h. The mixture was diluted with DMF (3 mL) and H_2O (1 mL), then degassed by bubbling nitrogen through the solution for 10 min. CuI (0.1 mg, 1.0 µmol, 0.1 eq.) and sodium ascorbate (0.1 µg, 1.0 µmol, 0.1 eq.) were added and the degassing was continued for 5 min. Sulfobetaine alkyne **6** (270 µg, 13.0 mmol, 2.00 eq.) was added and the mixture was stirred at 55 °C for 2 h. After addition of H_2O (20 mL) the solution was lyophilized to yield a dark blue lyophilizate. The crude product was purified *via* reversed phase flash chromatography (*loaded as solution* in H_2O (1 mL), 15 µm C18AQ silica, Interchim F0004 cartridge, 100% H_2O + 0.1% formic acid holding for 1 cv, then gradient to 100% MeCN + 0.1% formic acid in 15 cv, holding for 3 cv). After lyophilization Cy5-ZW-617 **9** (12 mg, 48%) was obtained as a dark blue lyophilizate.

$\boldsymbol{t_R}$ (Gravity SB, method 1) = 17.2 min.

HRMS (ESI) *m/z* $[M+2H]^{2+}$ calcd. for $C_{77}H_{104}N_{12}O_{20}S_3^{2+}$: 806.3321, found: 806.3331.

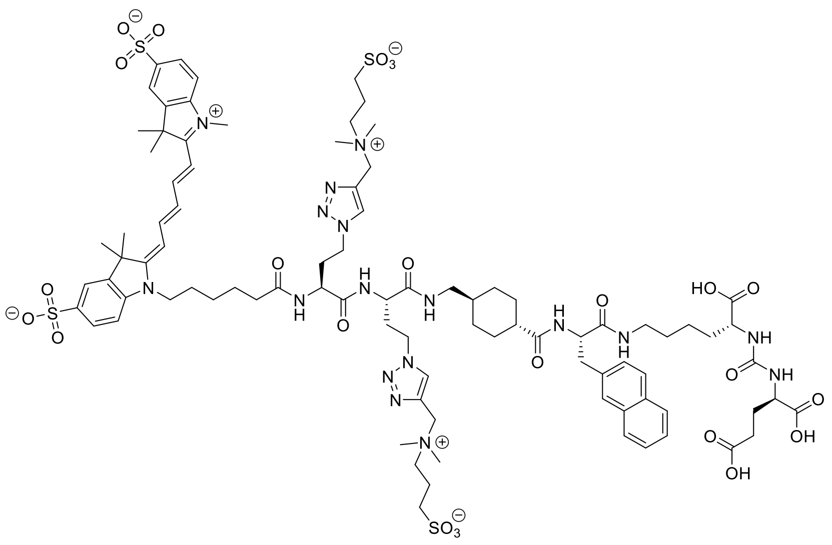

Cy5-ZW$_2$-617 (10). The same procedure as for Cy5-ZW-617 **9** was conducted with the modification that the coupling step with Fmoc-L-Aha-OH was repeated twice. The crude coupling product (600 µg, 7.00 µmol, 1.00 eq.) was dissolved in anhydrous DMF (1 mL), followed by addition of DIPEA (9.00 µL, 53.0 µmol, 8.00 eq.) and sulfo-Cy5 NHS ester **4** (4.90 mg, 7.00 µmol, 1.00 eq.). The resulting dark blue solution was stirred at room temperature under light exclusion for 12 h. The reaction mixture was diluted with DMF (3 mL) and H_2O (1 mL), then degassed by bubbling nitrogen through the solution for 10 min. CuI (0.1 mg, 1.0 µmol, 0.1 eq.) and sodium ascorbate (0.1 µg, 1.0 µmol, 0.1 eq.) were added and the degassing was continued for 5 min. Sulfobetaine alkyne **6** (540 µg, 26.0 mmol, 4.00 eq.) was added and the mixture was stirred at 55 °C for 2 h. After addition of H_2O (20 mL) the solution was lyophilized to yield a dark blue lyophilizate. The crude product was purified *via* reversed phase flash chromatography (*loaded as solution* in H_2O (0.5 mL), 15 µm C18AQ silica, Interchim F0004 cartridge, 100% H_2O + 0.1% formic acid holding for 1 cv, then gradient to 100% MeCN + 0.1% formic acid in 15 cv, holding for 3 cv). After lyophilization Cy5-ZW$_2$-617 **10** (8 mg, 62%) was obtained as a dark blue lyophilizate.

$\boldsymbol{t}_{\mathbf{R}}$ (Gravity SB, method 1) = 16.3 min.

HRMS (ESI) *m/z* $[M+2H]^{2+}$ calcd. for $C_{89}H_{125}N_{17}O_{24}S_4^{2+}$: 971.8978, found: 971.8958.

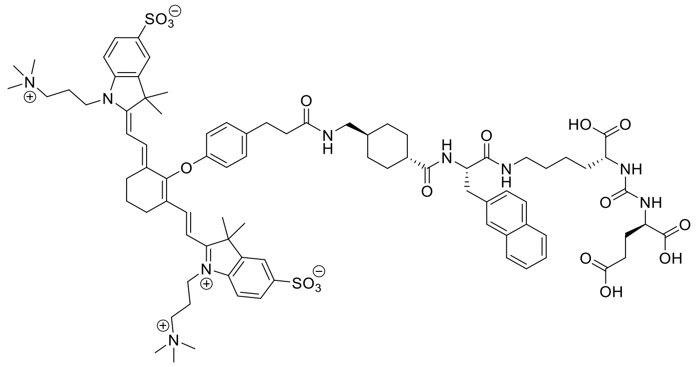

ZW800-617 (13). ZW800-1 **11** (5.4 mg, 6.0 µmol, 1.0 eq.) was dissolved in anhydrous DMSO (750 µL) followed by the addition of DIPEA (9.7 µL, 57 µmol, 10.0 eq.) and HATU (2.4 mg, 6.0 µmol, 1.1 eq.). The resulting dark green solution was stirred at room temperature for 10 min before a solution of the PSMA-617 entity **12** (7.5 mg, 11 µmol, 2.0 eq.). in anhydrous DMSO (250 µL) was added. The reaction solution was stirred at room temperature for 1 h and diluted with ice-cold isopropanol (8 mL) which resulted in precipitation of a dark green solid. The precipitate was collected by centrifugation, the colourless supernatant was discarded and the crude product was purified *via* reversed flash chromatography (*loaded as solution* in H_2O (0.3 mL), 15 µm C18AQ silica, Interchim F0004 cartridge, 100% H_2O + 0.1% formic acid holding for 1 cv, then gradient to 100% MeCN + 0.1% formic acid in 15 cv, holding for 3 cv). After lyophilization ZW800-617 **13** (5 mg, 55%) was obtained as a dark green lyophilizate.

t_R (Gravity SB, method 1) = 12.8 min.

HRMS (ESI) *m/z* $[M+H]^{2+}$ calcd. for $C_{84}H_{111}N_9O_{17}S_2^{2+}$: 790.8764, found: 790.8767.

Analytical Data

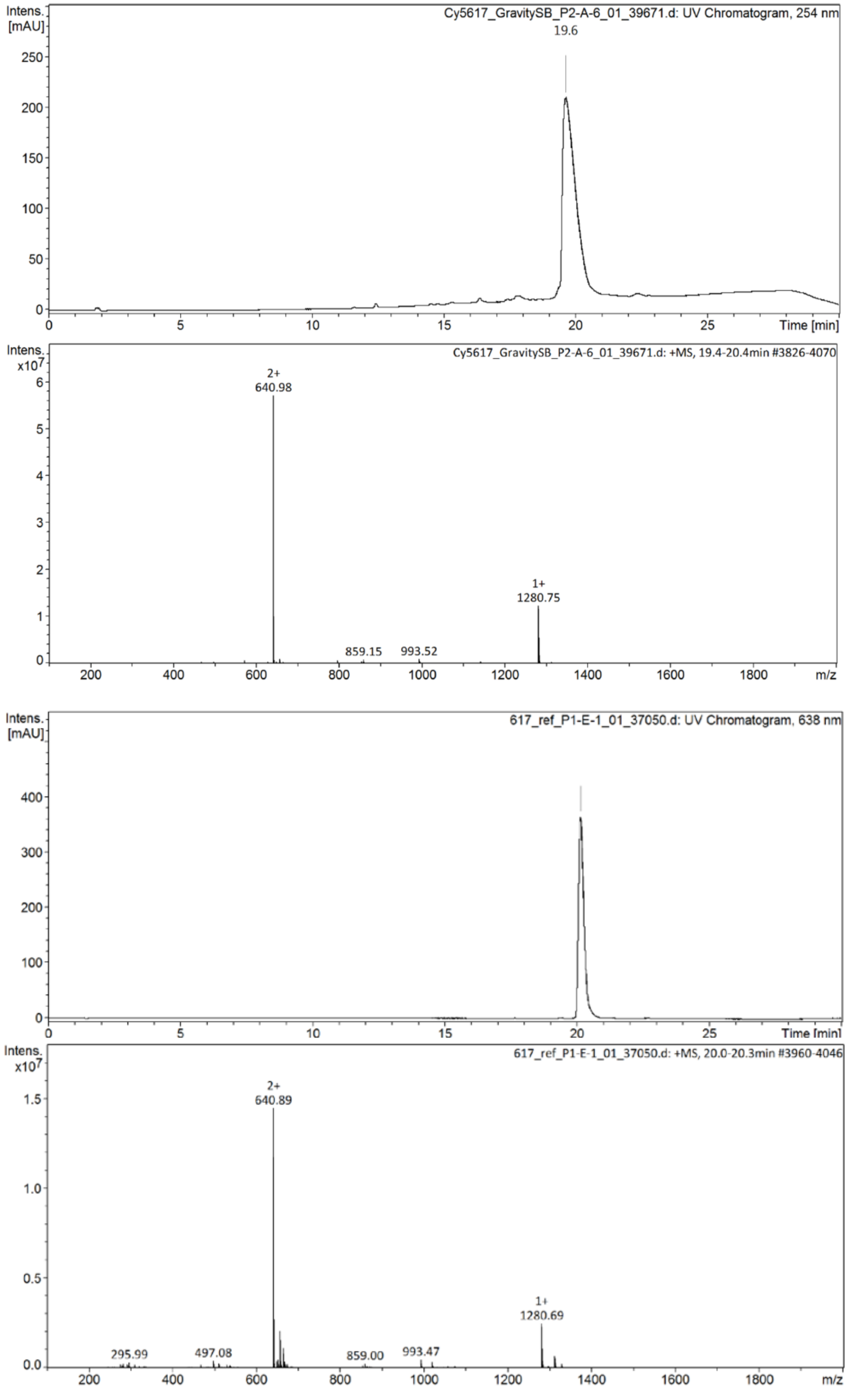

Figure S8: HPLC-MS) chromatograms (MN C18 Gravity-SB, method 1, λ = 254 and 638 nm) of Cy5-617 **8** (Purity$_{254\ nm}$ > 98%).

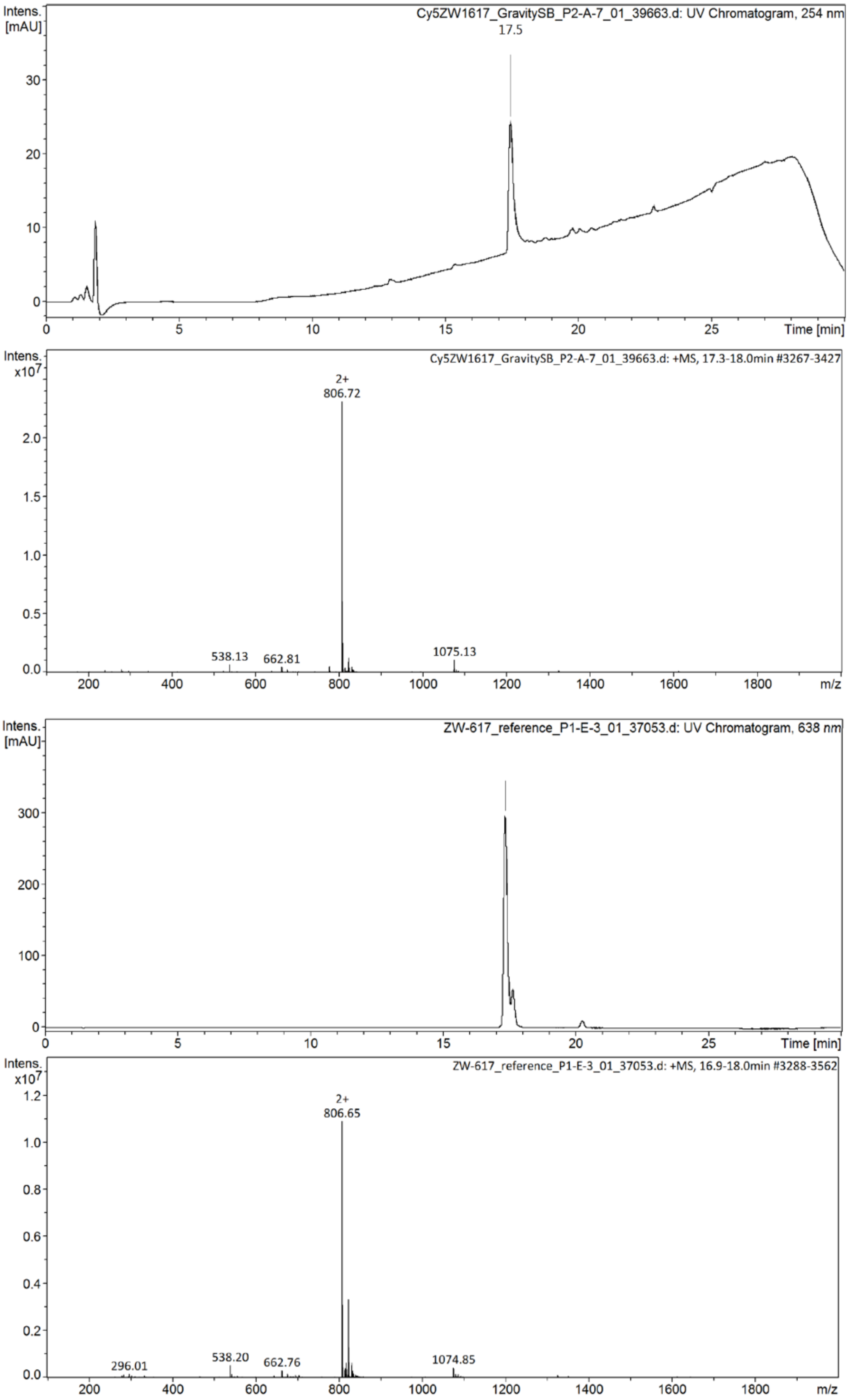

Figure S9: HPLC-MS (MN C18 Gravity-SB, method 1, λ = 254 and 638 nm) chromatogram of Cy5-ZW-617 **9** (Purity$_{254\ nm}$ > 96%)

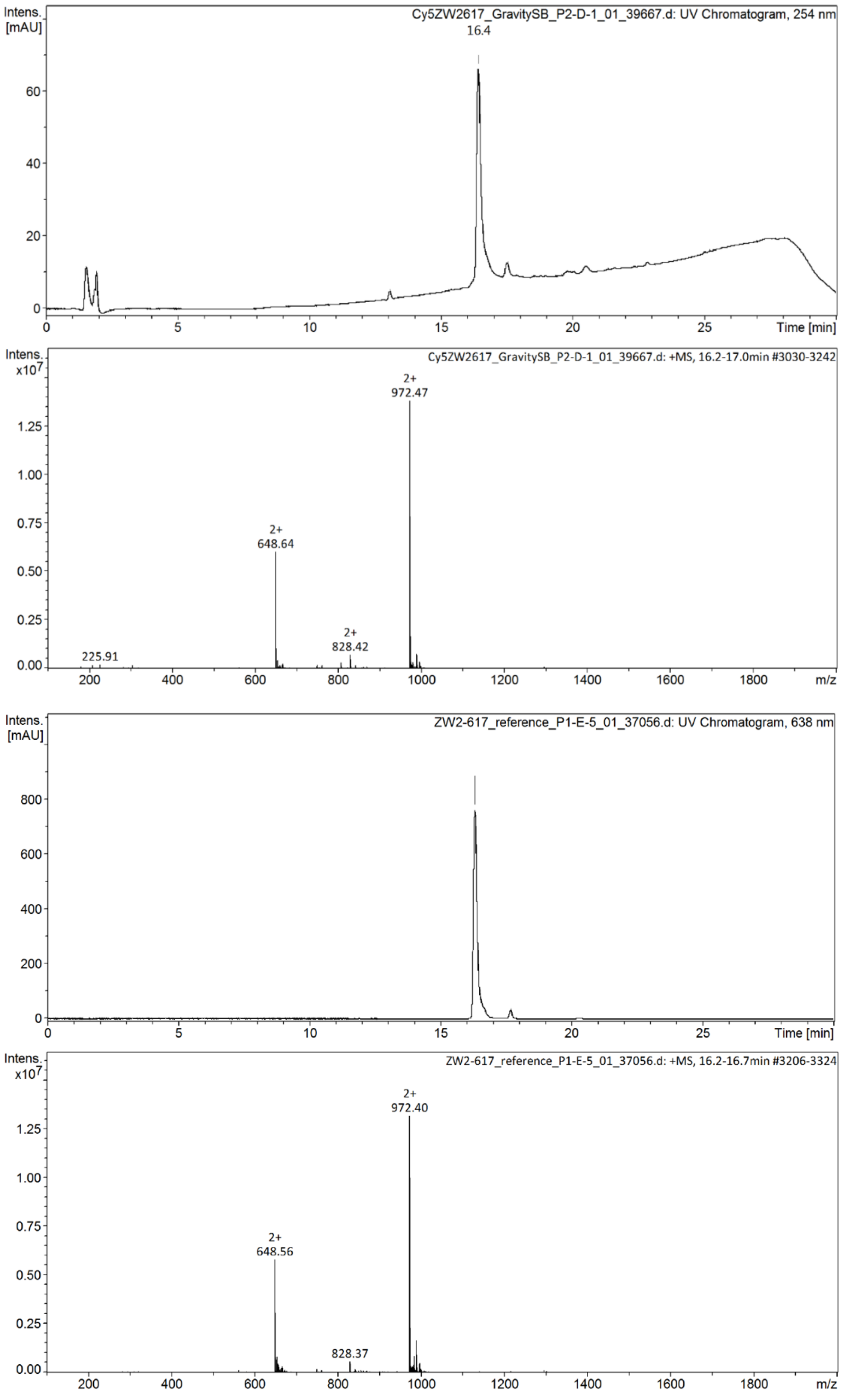

Figure S10: HPLC-MS (MN C18 Gravity-SB, method 1, λ = 254 and 638 nm) chromatogram of Cy5-ZW$_2$-617 **10** (Purity$_{254\ nm}$ > 95%)

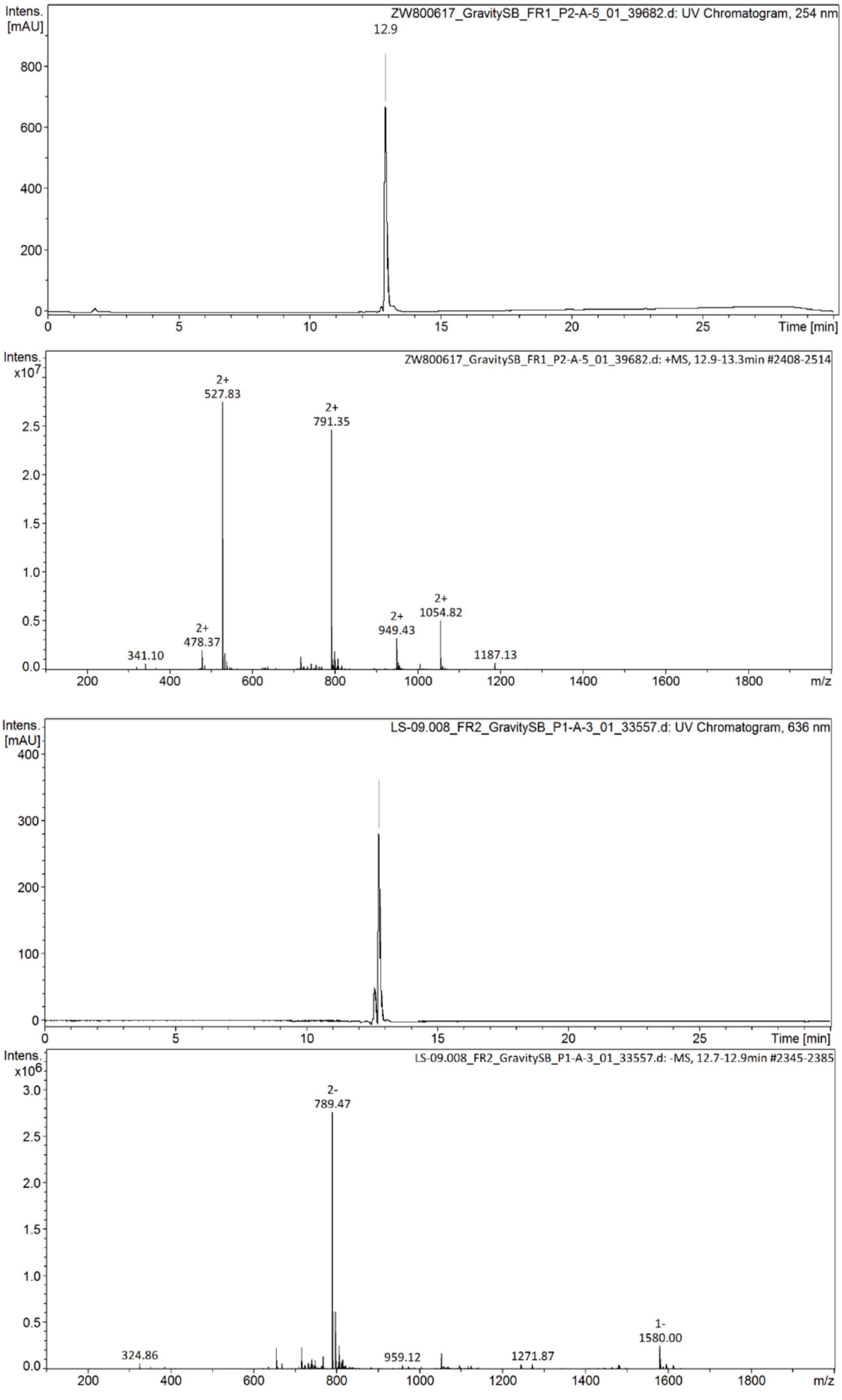

Figure S11: HPLC-MS (MN C18 Gravity-SB, method 1, λ = 254 and 638 nm) chromatogram of ZW800-617 **13** (Purity$_{254\ nm}$ > 98%).

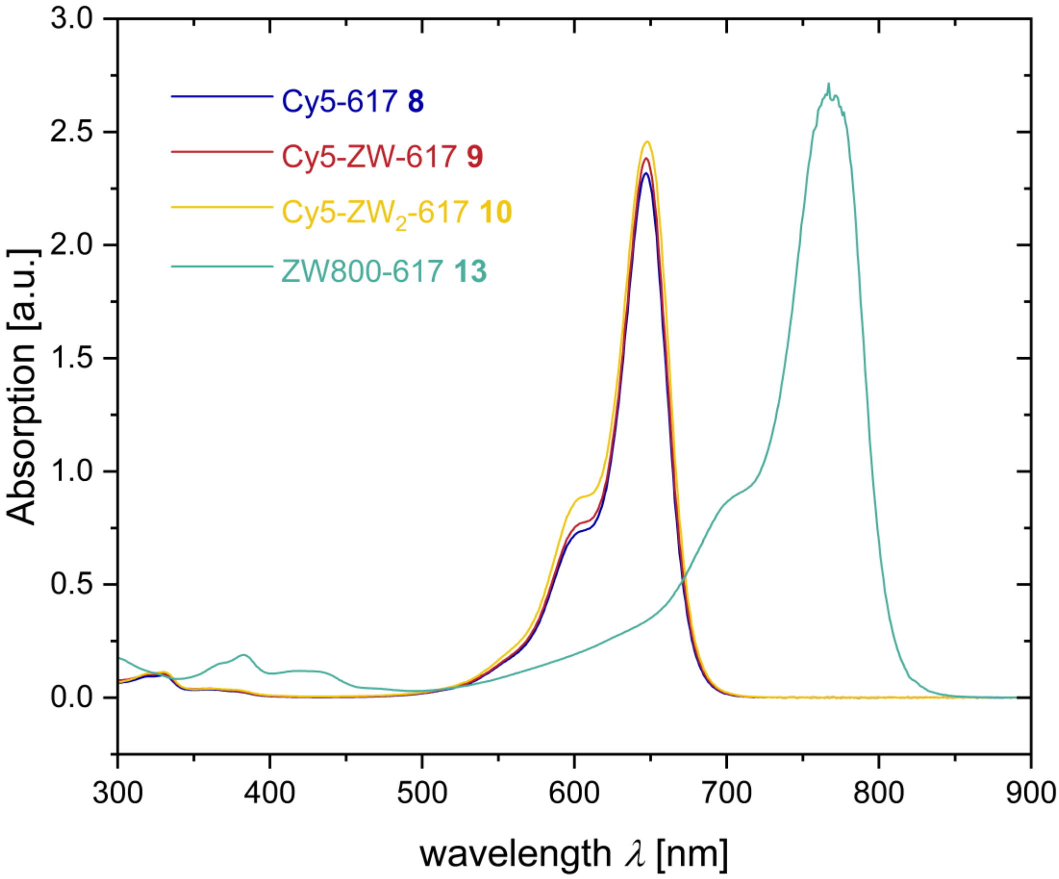

Figure S12: UV-vis absorption spectra of Cy5 dye conjugates **8**, **9**, **10** and the ZW800 conjugate **13** in aqueous PBS buffer (pH 7.4).

Table S3. Physicochemical properties, binding affinity and uptake metrics of the dye conjugates.

**Compound**	**IC50 [nM][a]**	**log$D_{7.4}$ [b]**	**t_R (HPLC) [min]**	**Rel. HSA binding**	**Residual body dose [nmol][c][d]**
PSMA-617	2.41 ± 0.09	−4.4 (lit.)[1]	-	-	-
Cy5-617 **8**	4.09 ± 2.96	−1.16 ± 0.02	20.1	60.3 ± 2.6%	1.9
Cy5-ZW-617 **9**	6.26 ± 3.66	−1.14 ± 0.01	17.2	26.9 ± 2.2%	0.8
Cy5-ZW_2-617 **10**	4.39 ± 1.69	−1.27 ± 0.06	16.3	15.3 ± 2.0%	0.25
ZW800-617 **13**	4.46 ± 1.75	−1.92 ± 0.17	12.8	16.3 ± 4.1%	1.0

[a] Determined *via* NAALADase assay (rhPSMA); [b] *n*-octanol / PBS, pH 7.4; [c] 4 h p.i. of 20 nmol; [d] PSMA-negative tumor-bearing MMTV-PyMT mice.

References

(1) Umbricht, C. A.; Benesova, M.; Schibli, R.; Muller, C. Preclinical Development of Novel PSMA-Targeting Radioligands: Modulation of Albumin-Binding Properties To Improve Prostate Cancer Therapy. *Mol. Pharm.* **2018**, *15*, 2297-2306. DOI: 10.1021/acs.molpharmaceut.8b00152
